# Genome-wide association reveals QTL for growth, bone and in vivo carcass traits as assessed by computed tomography in Scottish Blackface lambs

**DOI:** 10.1186/s12711-016-0191-3

**Published:** 2016-02-08

**Authors:** Oswald Matika, Valentina Riggio, Marie Anselme-Moizan, Andrew S. Law, Ricardo Pong-Wong, Alan L. Archibald, Stephen C. Bishop

**Affiliations:** The Roslin Institute and R(D)SVS, University of Edinburgh, Edinburgh, UK; INP, Ecole Nationale Vétérinaire, Toulouse, France

## Abstract

**Background:**

Improving meat quality including taste and tenderness is critical to the protection and development of markets for sheep meat. Phenotypic selection for such measures of meat quality is constrained by the fact that these parameters can only be measured post-slaughter. Carcass composition has an impact on meat quality and can be measured on live animals using advanced imaging technologies such as X-ray computed tomography (CT). Since carcass composition traits are heritable, they are potentially amenable to improvement through marker-assisted and genomic selection. We conducted a genome-wide association study (GWAS) on about 600 Scottish Blackface lambs for which detailed carcass composition phenotypes, including bone, fat and muscle components, had been captured using CT and which were genotyped for ~40,000 single nucleotide polymorphisms (SNPs) using the Illumina OvineSNP50 chip.

**Results:**

We confirmed that the carcass composition traits were heritable with moderate to high (0.19–0.78) heritabilities. The GWAS analyses revealed multiple SNPs and quantitative trait loci (QTL) that were associated with effects on carcass composition traits and were significant at the genome-wide level. In particular, we identified a region on ovine chromosome 6 (OAR6) associated with bone weight and bone area that harboured SNPs with p values of 5.55 × 10^−8^ and 2.63 × 10^−9^, respectively. The same region had effects on fat area, fat density, fat weight and muscle density. We identified plausible positional candidate genes for these OAR6 QTL. We also detected a SNP that reached the genome-wide significance threshold with a *p* value of 7.28 × 10^−7^ and was associated with muscle density on OAR1. Using a regional heritability mapping approach, we also detected regions on OAR3 and 24 that reached genome-wide significance for bone density.

**Conclusions:**

We identified QTL on OAR1, 3, 24 and particularly on OAR6 that are associated with effects on muscle, fat and bone traits. Based on available evidence that indicates that these traits are genetically correlated with meat quality traits, these associated SNPs have potential applications in selective breeding for improved meat quality. Further research is required to determine whether the effects associated with the OAR6 QTL are caused by a single gene or several closely-linked genes.

**Electronic supplementary material:**

The online version of this article (doi:10.1186/s12711-016-0191-3) contains supplementary material, which is available to authorized users.

## Background

It is generally considered that the sheep meat industry cannot compete with poultry and pig meat industries both in volume and price but should focus on quality and consistency [1]. For lamb production to improve or maintain its competitiveness in the meat market place, consumers need assurances in terms of healthiness, composition and taste of the product. Improvements in product quality rely on the identification of measurable carcass composition and meat quality traits. One of the factors that determine meat quality is fat content, and thus, the public’s perception of animal fat and associated health risks including increased risks of cardiovascular diseases will also have impacts on selective breeding goals [2, 3].

Phenotypic selection for meat quality parameters, including taste and tenderness, is constrained by the fact that they can only be measured post-slaughter. However, these meat quality parameters are influenced by carcass composition [4], including the level and distribution of fat, which can be measured on live animals. Before considering measures on live animals, it should be noted that dissection is often regarded as the gold standard for assessing carcass composition but this is a costly, laborious and time-consuming process with the possibility of human errors in the measurements and can only be undertaken post-slaughter [5]. Similarly, measuring the chemical composition of the carcass including components such as nitrogen (protein), lipids (fat), ash and water, can only performed post-mortem. Live-weight is recognized as an effective measure of body mass, but is a poor indicator of body composition [6]. However, there are a number of methods for measuring the composition of lamb carcasses that range from visual classification, linear measurements using human or computer vision, infrared (IR) or near infrared (NIR) reflectance, conductivity, bio-impedance to computer tomography (CT) [7]. Technologies such as CT offer the possibility of non-invasive and accurately measured carcass traits on live animals. Junkuszew and Ringdorfer [8] reported correlations of 0.88 or 0.72 and 0.68 or 0.48 between actual fat in the carcass and fat and muscle contents, which were measured by CT or ultrasonography (US), respectively. Measurements were taken between the 5th and 6th thoracic vertebra and between the 10th and 11th thoracic vertebra. Most studies that compare CT measurements/predictions of in vivo measurements to other methods of carcass prediction have demonstrated that CT estimates were the most accurate [9–15]. Although CT predictions are usually better predictors of carcass composition, other factors, such as cost and ease of use, restrict its use [7].

Previous studies identified that carcass composition traits assessed by CT are heritable and that they are correlated with both growth and meat quality traits [16, 17]. Quantitative trait loci (QTL) for both carcass composition and meat quality have been reported in the literature based on microsatellite markers [6, 16, 18, 19]. The aim of our study was to exploit improved genotyping tools and, specifically, the Illumina OvineSNP50 SNP chip that allows the simultaneous characterization of up to 54,241 independent single nucleotide polymorphisms (SNPs), to identify QTL and potential candidate genes with effects on carcass composition measured by CT.

## Methods

### Data description

Data were available for 751 pedigree-recorded Scottish Blackface lambs born between 2001 and 2003 from a flock that originated from previously selected fat and lean lines, established in 1988 [20].

*In vivo* carcass composition measurements (on 600 lambs) were obtained once a year, over 3 days at an average age of 24 weeks using CT. Cross-sectional scans were taken at the ischium (ISC), the 5th lumbar vertebra (LV5) and the 8th thoracic vertebra (TV8), and from each scan image, the areas and image densities were obtained for fat, muscle and bone components of the carcass. Live weight (LW) was also recorded at the time of the CT measurements. Prediction equations for total weight of each tissue were obtained from a calibration study [Scotland’s Rural College (SRUC) formerly known as Scottish Agricultural College (SAC), unpublished] on unrelated Scottish Blackface lambs of the same age, and are as follows:

Fat weight = −1340 + (63.6 × LW) + (0.351 × fat area LV5) + (0.248 × fat area TV8), (R^2^ = 91.9 %);

Bone weight = −102 + (39.1 × LW) + (0.241 × bone area ISC) + (0.762 × bone area LV5), (R^2^ = 73.5 %);

Muscle weight = −1640 + (87.3 × LW) + (0.242 × muscle area ISC) + (0.163 × muscle area TV8), (R^2^ = 84.8 %);

Total carcass weight = fat weight + bone weight + muscle weight;

Fat (or bone or muscle) proportion as fat (or bone or muscle) weight over total carcass weight.

These prediction equations were used to calculate the following traits: predicted fat weight, predicted muscle weight, predicted bone weight, predicted total carcass weight, fat proportion, muscle proportion, bone proportion, fat, muscle and bone areas at the cross sectional scan of the ischium (mm^2^) and their densities (see Karamichou et al. [16] for more details). We also derived the muscle to bone ratio (M:B) from muscle weight divided by bone weight and killing out proportion (KO) obtained by dividing total carcass weight by LW.

All animals were genotyped with the Illumina OvineSNP50 SNP chip, which allows the simultaneous characterization of up to 54,241 independent SNPs. After quality control (QC), which removed SNPs with a minor allele frequency (MAF) less than 0.05 and SNPs with a call rate less than 0.90, 40,264 SNPs remained for further analyses. SNP positions were obtained from the Sheep Genome browser v3.0 (http://www.livestockgenomics.csiro.au/sheep/).

### Statistical analyses

Genetic parameter estimates were obtained using ASReml [21] by fitting the fixed effects of sex (male and female), year (2001 to 2003), management group (two levels), litter size (single or twins), age of dam (1 to 4+ years), line (1 to 7) and with effects of dates of birth and slaughter as covariates. The distributions of trait data values were checked for normality. The animal was fitted as a random effect by using either the complete available pedigree or the genomic relationship matrix.

The single SNP association analysis was performed using the GenABEL package [22] in R environment (http://www.r-project.org). In this analysis, because of the previous selection history of the flock, it was important to identify and correct for population stratification. This was done by using classical multi-dimension scaling (MDS) to explore population substructure and to verify the genetic homogeneity of the sample prior to analysis. To account for relatedness, the variance/covariance matrix was estimated from the genomic kinship matrix that was constructed by using pair-wise identities by state, and calculated for all samples based on all autosomal SNPs, as implemented in the GenABEL library [22]. First, both fixed and polygenic effects for the traits were considered with the latter accounting for genetic relationships between animals. The fixed effects considered were as described above, with the first three principal components (PC) used as a substitute of the effects of line. Second, associations were tested using a mixed model function, i.e. an mmscore function [23] on the residuals, which were corrected for relatedness. After Bonferroni correction, the p values for the genome-wide (p < 0.05) and the suggestive (i.e., one false positive SNP out of all 40,264 SNPs) significance thresholds were less than 1.24 × 10^−6^ and less than 2.48 × 10^−5^, respectively. The p values were corrected for the genomic inflation factor λ, which takes into account population substructure.

After the GWAS analyses with the OvineSNP50 SNP chip, the top four significant SNPs, with the lowest p value for each trait, were further tested individually for association in ASReml with the following traits: fat weight (fat_wt) (g), muscle weight (mus_wt) (g), bone weight (bon_wt) (g), carcass total weight (cs_tot_wt) (g), live weight (LW) (kg), killing out proportion (KO_P), fat proportion (f_P), muscle proportion (m_P), bone proportion (b_P), muscle to bone ratio (M:b), fat area at the ischium (fat_area_ISC) (mm^2^), fat density at ISC (fat_density_ISC), muscle area at the ISC (mus_area_ISC) (mm^2^), muscle density at the ISC (mus_density_ISC), bone area at the ISC (bon_area_ISC) (mm^2^), bone density at the ISC (bon_density_ISC), fat area at the ISC accounting for live weight (fat_area_ISC_LW) (mm^2^), fat area at 5th lumbar vertebrae (fat_area_LV5) (mm^2^), fat density fat_density_LV5, muscle area at 5th lumbar vertebrae (mus_area_LV5) (mm^2^), muscle density at the LV5 (mus_density_LV5), bone area at LV5 (bon_area_LV5) (mm^2^), bone density at LV5 (bon_density_LV5), fat area at LV5 accounting for LW (fat_area_LV5_LW) (mm^2^), fat area at 8th thoracic vertebrae (fat_area_TV8) (mm^2^), fat density at TV8 (fat_density_TV8), muscle area at TV8 (mus_area_TV8) (mm^2^), muscle density at TV8 (mus_density_TV8), bone area at TV8 (bon_area_TV8) (mm^2^), bone density at TV8 (bon_density_TV8), fat area at TV8 accounting for LW (fat_area_TV8_LW) (mm^2^). The SNPs were fitted as fixed effects (see model used for association that fits the **A** matrix). These analyses also enabled us to estimate the additive and dominance effects of each SNP. With AA, BB and AB defined as the predicted trait values for each genotype class, p and q the SNP allele frequencies and VA the total additive genetic variance of the trait obtained when no SNP effects are included in the model, genetic effects were then calculated as follows: additive effect, a = (AA − BB)/2; dominance effect, d = AB − [(AA + BB)/2]; and proportion of genetic variance due to SNP = [2pq(a + d(q − p))2]/VA.

Finally, the data were analysed using the regional genomic relationship mapping or regional heritability mapping (RHM) approach [24], in which each ovine chromosome (OAR for *Ovies aries* chromosome) is divided into windows of a pre-defined number of SNPs, and the variance attributable to each window is estimated. A window size of 100 adjacent SNPs was used, and the window was shifted every 50 SNPs. For the analysis, we used a mixed model that accounted for the same fixed effects as those previously fitted in the GenABEL analysis. The residual and additive genetic (both region-specific and whole-genome) effects were fitted as random effects. The whole-genome additive effect was estimated using the genomic relationship matrix constructed from all SNPs, whereas the region-specific additive effect was estimated from a genomic relationship matrix constructed from the SNPs within each window, i.e. region. Whole-genome, region-specific and residual variances defined as $\upsigma_{\rm{a}}^{2}$, $\upsigma_{\rm{w}}^{2}$ and $\upsigma_{\rm{e}}^{2}$, respectively, the phenotypic variance, $\upsigma_{\rm{p}}^{2}$, is then given by $\upsigma_{\rm{a}}^{2} + \upsigma_{\rm{w}}^{2} + \upsigma_{\rm{e}}^{2}$. Whole-genome heritability was estimated as $\rm{h}_{\rm{a}}^{2} = {{\left( {\upsigma_{\rm{a}}^{2} + \upsigma_{\rm{w}}^{2} } \right)} / {\upsigma_{\rm{p}}^{2} }}$ whereas the region-specific heritability was $\rm{h}_{\rm{w}}^{2} = {{\upsigma_{\rm{w}}^{2} } / {\upsigma_{\rm{p}}^{2} }}$.

To test for differences in region-specific variance, a likelihood ratio test (LRT) was used to compare a model that fitted variance within a specific window (fitting both whole-genome and region-specific additive variance) against the null hypothesis of no variance in that window (whole-genome additive variance only). The test statistic was assumed to follow a $1/2 \chi_{(1)}^{2}$ distribution [25]. In total, 858 windows were tested across chromosomes, of which half were used in the Bonferroni correction to account for the sliding windows. Hence, after Bonferroni correction to account for multiple-testing, the LRT thresholds were equal to 13.54 and 9.27, corresponding to p values less than 1.17 × 10^−4^ and less than 2.33 × 10^−3^, for genome-wide and suggestive significance levels, respectively.

The most significant region identified in the study was explored for linkage disequilibrium (LD) with data prepared using Plink software v1.07 [26]; calculation and visualisation of haplotype blocks and LD were achieved using Haploview v4 software [27]. LD was calculated as a percentage of allele frequencies using r^2^ or D′.

## Results

### Estimated genetic parameters

Heritability estimates for bone traits were generally higher when estimated by using the pedigree matrix rather than the kinship matrix. These estimates ranged from moderate to high values (0.14 to 0.60 when using the kinship matrix and 0.14 to 0.68 when using the pedigree matrix [See Additional file 1: Table S1]). However, heritability estimates for muscle traits were lower when using the kinship matrix (0.27 to 0.61) than when using the pedigree matrix (0.41 to 0.61) [See Additional file 1: Table S2]. In contrast, heritability estimates for fat traits ranged from low to high values (0.20 to 0.77) using the kinship matrix and (0.02 to 0.97) using the pedigree matrix [See Additional file 1: Table S3]. In general, estimates obtained by fitting the pedigree matrix were higher than those obtained by fitting the kinship matrix except for fat density measured at the ischium and accounting for LW [See Additional file 1: Table S3]. Finally, heritability estimates for proportions of fat, bone and muscle, total carcass weight and LW were moderate to high (0.32 to 0.47) using the kinship matrix and high (0.55 to 0.70) using the pedigree matrix [See Additional file 1: Table S4].

### Single SNP association study

**Fig. 1:**
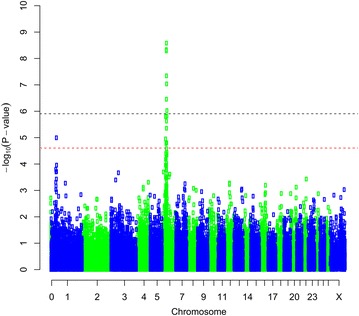
Manhattan plot of the GWA results [−log_10_(p) of the corresponding p values corrected for the genomic inflation factor λ] for bone area at the ischium. Genome-wide (p < 0.05) threshold is represented as a *dashed black line* and suggestive threshold as a *dashed red line*

**Fig. 2:**
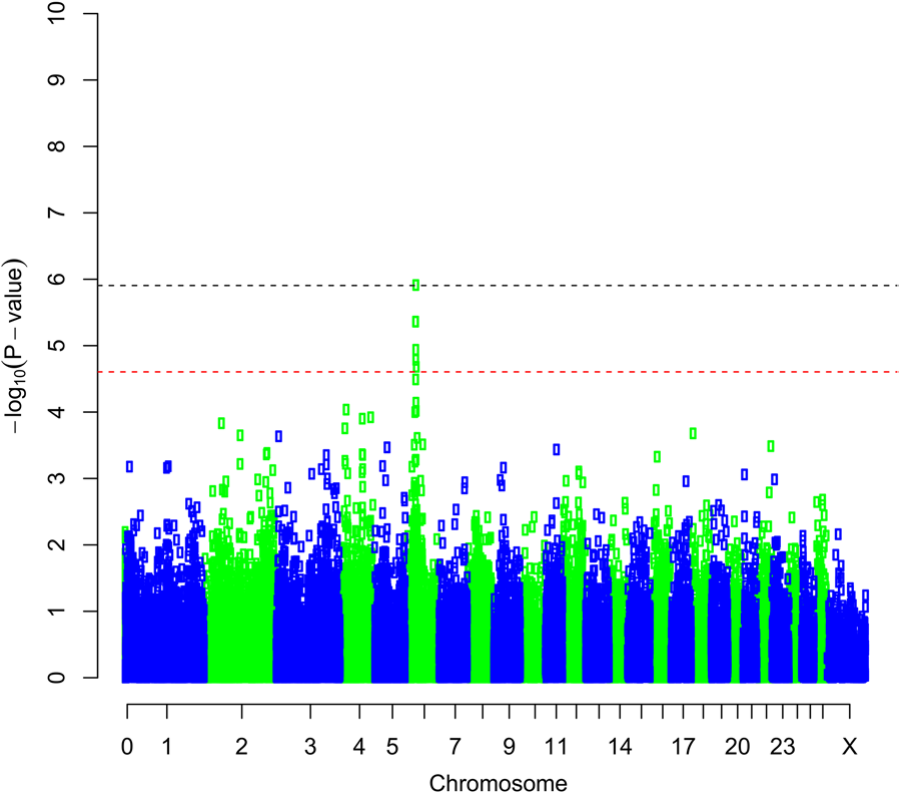
Manhattan plot of the GWA results [−log_10_(p) of the corresponding p values corrected for the genomic inflation factor λ] for fat weight accounting for live weight. Genome-wide (p < 0.05) threshold is represented as a *dashed black line* and suggestive threshold as a *dashed red line*

**Fig. 3:**
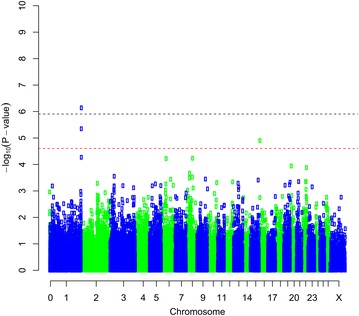
Manhattan plot of the GWA results [−log_10_(p) of the corresponding p values corrected for the genomic inflation factor λ] for muscle density at the 8th thoracic vertebra. Genome-wide (p < 0.05) threshold is represented as a *dashed black line* and suggestive threshold as a *dashed red line*

The association analysis using GenABEL identified SNPs that reached the genome-wide significance threshold (p < 0.05) for the following four traits: bone area at ISC, bone weight, fat weight accounting for LW and muscle density at TV8 (Table 1). Three of the traits (i.e. bone area at ISC (Fig. 1), bone weight [See Additional file 2: Figure S1] and fat weight accounting for LW (Fig. 2) were associated with SNPs on OAR6 with p values of $2.63 \times 10^{ - 9}$, $5.55 \times 10^{ - 8}$ and $1.23 \times 10^{ - 6}$, respectively. Interestingly, there was also a region on OAR6 with effects on fat area at TV8 when accounting for LW (Table 1). The region associated with muscle density at TV8 was located on OAR1 with highest SNP having a p value of $7.28 \times 10^{ - 7}$ (Fig. 3). Other suggestive QTL were observed for bone proportion and killing out percentage (Table 1). Selected QQ plots are in Additional file 3: Figures S2, S3, S4, S5, S6, S7, and S8.

**Table 1:** Significant SNPs identified in the association analysis using GenABEL

	Chromosome	Position	p Value^a^
Bone area at the ischium			
OAR6_40855809	6	36655091	2.63E-09
OAR6_40277406	6	36073405	4.66E-09
OAR6_40311379	6	36104954	5.19E-09
DU178311_404	6	36839547	4.58E-08
OAR6_42576838	6	38310652	9.24E-08
OAR6_40955920	6	36750972	3.52E-07
OAR6_45662944	6	40975597	9.51E-07
OAR6_45285663	6	40594243	1.53E-06
s71628	6	29009593	1.59E-06
OAR6_41558126	6	37334387	2.36E-06
OAR6_41424992	6	37203894	4.43E-06
OAR1_50287726	1	48393355	1.02E-05
OAR6_32516276	6	28676510	1.12E-05
OAR6_43292772	6	38906528	1.57E-05
OAR6_37176737	6	33197652	1.69E-05
Bone weight			
OAR6_40855809	6	36655091	5.55E-08
OAR6_40277406	6	36073405	1.12E-06
OAR6_40311379	6	36104954	1.46E-06
OAR6_41558126	6	37334387	5.75E-06
OAR6_37176737	6	33197652	1.49E-05
OAR6_45662944	6	40975597	2.06E-05
OAR6_41424992	6	37203894	2.16E-05
s17946	6	37164263	2.34E-05
Fat area at the 8th thoracic vertebra accounting for live weight			
OAR6_40955920	6	36750972	3.37E-06
DU178311_404	6	36839547	3.43E-06
OAR6_40311379	6	36104954	4.94E-06
OAR6_40277406	6	36073405	5.57E-06
Fat weight accounting for live weight			
DU178311_404	6	36839547	1.23E-06
OAR6_40311379	6	36104954	4.37E-06
OAR6_40277406	6	36073405	4.38E-06
OAR6_40855809	6	36655091	1.16E-05
OAR6_40955920	6	36750972	1.65E-05
OAR6_42576838	6	38310652	2.10E-05
Muscle density at the 8th thoracic vertebra			
s66995	1	275593974	7.28E-07
s45224	1	275482364	4.50E-06
s45240	16	5438002	1.26E-05
Killing out percentage			
DU178311_404	6	36839547	1.61E-05
Bone proportion			
s75746	3	69399949	3.89E-06

^a^The genome-wide significance threshold corresponded to a p value less than 1.24 × 10^−6^ and the suggestive significance threshold corresponded to a p value less than 2.48 × 10^−5^

When fitted individually, the most significant SNPs associated with bone area at ISC and bone weight (OAR6_40855809 on OAR6), fat weight accounting for LW (DU178311_404 on OAR6), fat area at TV8 accounting for LW (OAR6_40955920 on OAR6) and muscle density at TV8 (s66995 on OAR1), associated (p < 0.05, nominal p value) with more traits than those identified in the previous GWAS analysis [See Additional file 4: Table S5]. Minor allele frequencies for all four SNPs ranged from 0.33 to 0.37. The summary statistics for allele substitution effects, dominance effects, proportions of genetic and phenotypic variances explained by these SNPs, and proportions of both genetic and phenotypic deviations explained by these SNPS are in Additional file 4: Tables S6, S7, S8 and S9. The traits with the most significant p values were bone area at ISC and bone weight with p values of $8.12 \times 10^{ - 10}$ and $7. 7 9\times 1 0^{ - 9} ,$ respectively [See Additional file 4: Table S6] for the SNP, “OAR6_40855809”. The allele substitution effect was 1.4 ± 0.29 kg for LW to 100 ± 16.5 g for bone weight. Only one trait, fat density at ISC, had significant dominance effects. The proportion of additive genetic variance explained by this SNP varied from 0 (when there was dominance) to 55 % for bone area at ISC. Similarly, the genetic standard deviations explained by this SNP ranged from 0 to 1.05 for bone area at ISC [See Additional file 4: Table S6]. We observed similar trends for SNP DU178311_404 and OAR6_40955920 [See Additional file 4: Tables S7 and S8]. Dominance effects were significant (p < 0.05) for muscle area at ISC and at LV5 for SNP s66995, however, muscle density at LV5 and TV8 had significant additive genetic effects [See Additional file 4: Table S9].

### Regional heritability mapping

**Fig. 4:**
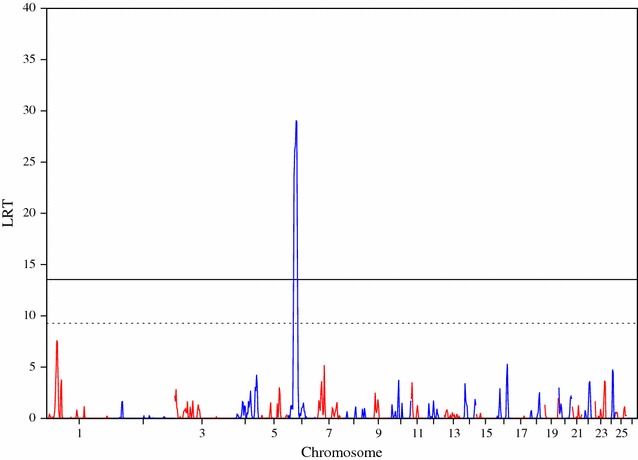
Manhattan plot of the LRT across the genome for bone area at ischium, using regional heritability mapping. Genome-wide threshold (p < 0.05) is represented as a *solid line* and suggestive threshold as a *dashed line*

Regional heritability mapping (RHM) identified more genome-wide significant regions (p < 0.05) than the association analyses. For bone traits, the most significant region was identified on OAR6 specifically for bone weight accounting for LW, bone weight at ISC, and bone weight at ISC accounting for LW (Table 2). An example of the genome-wide RHM Manhattan plot for bone traits that reached the genome-wide threshold, and specifically for bone area at ISC, is in Fig. 4 and the plots for the other bone traits are in Additional file 5: Figures S9, S10, S11, S12 and all plots with a suggestive threshold for bone traits are in Additional file 6: Figures S13, S14, S15, S16 and S17. Other genome-wide regions (p < 0.05) for bone traits were identified on OAR24 for bone density at LV5 accounting for LW and OAR3 for bone density at TV8 (Table 2) with corresponding Manhattan plots in Figures S4 and S5 [See Additional file 5: Figures S11 and S12].

**Table 2:** Regional heritability (h2w) for bone traits, for windows of the genome that were significant at both the genome-wide (p < 0.05) and suggestive levels

	Chr	Windows	Start_position	End_position	LRT^a^	H2w
Bone weight	6	12	30301209	36839547	9.38	0.05
	6	13	33493183	39305012	12.46	0.06
	6	14	36914376	41901628	11.34	0.06
	19	1	286449	6216575	9.94	0.06
Bone weight accounting live weight	6	6	15078062	20110817	9.88	0.09
	6	7	17621458	22303956	10.06	0.08
	6	10	24995206	30283083	9.52	0.08
	6	11	27872427	33467474	14.82	0.13
	6	12	30301209	36839547	15	0.07
	6	13	33493183	39305012	12.06	0.05
	6	14	36914376	41901628	11.14	0.05
Bone area at ischium	6	9	22323737	27784139	10.92	0.11
	6	10	24995206	30283083	23.68	0.12
	6	11	27872427	33467474	26.2	0.13
	6	12	30301209	36839547	26.6	0.10
	6	13	33493183	39305012	29.06	0.08
	6	14	36914376	41901628	28.94	0.08
	6	15	39327441	44214759	14.94	0.07
Bone area at ischium accounting for live weight	6	10	24995206	30283083	18.48	0.09
	6	11	27872427	33467474	21.1	0.09
	6	12	30301209	36839547	21	0.08
	6	13	33493183	39305012	20.68	0.06
	6	14	36914376	41901628	21.32	0.06
	6	15	39327441	44214759	11.48	0.06
Bone area at 5th lumbar vertebra accounting for live weight	6	5	12560057	17588020	9.14	0.08
	6	6	15078062	20110817	11.38	0.10
	6	7	17621458	22303956	11.6	0.08
Bone density at ischium	7	28	73086813	78504393	9.44	0.10
	7	29	75173621	81259598	10.12	0.10
Bone density at ischium accounting for live weight	6	13	33493183	39305012	12.76	0.05
	11	16	50499706	57193316	9.16	0.06
Bone density at 5th lumbar vertebra	12	9	23152711	28032557	10.94	0.07
	12	10	25524368	30167375	10.56	0.08
	24	7	20406106	28037384	9.92	0.08
	24	8	24040495	31229367	15.98	0.11
Bone density at 8th thoracic vertebra	3	24	64458505	69730945	12.18	0.09
	3	25	66881852	72060440	11.9	0.06
	3	26	69775020	75107095	9.62	0.07
	22	2	4058030	8888071	9.4	0.08
Bone density at 8th thoracic vertebra accounting for live weight	3	23	61850473	66786673	9.18	0.09
	3	24	64458505	69730945	16.04	0.13
	3	25	66881852	72060440	14.9	0.08
	3	26	69775020	75107095	13.58	0.09

*Chr* chromosome

^a^The genome-wide LRT threshold was equal to 13.54, corresponding to a p value less than 1.17 × 10^−4^ and the suggestive LRT threshold was equal to 9.27, corresponding to a p value less than 2.33 × 10^−3^

**Fig. 5:**
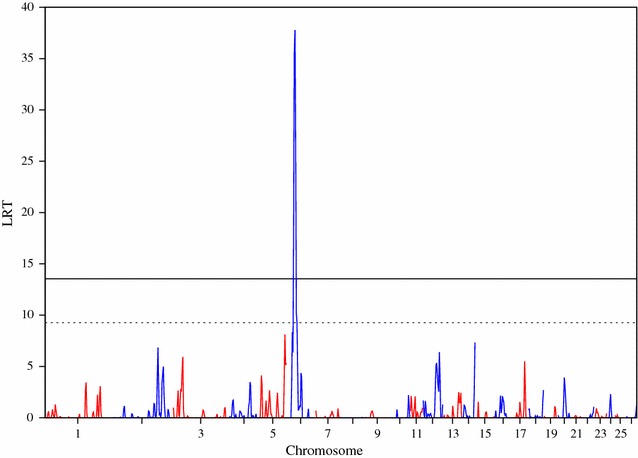
Manhattan plot of the LRT across the genome for fat weight accounting for live weight, using regional heritability mapping. Genome-wide threshold (p < 0.05) is represented as a *solid line* and suggestive threshold as a *dashed line*

Four fat traits were above the genome-wide threshold (p < 0.05) with large LRT values of 37.78, 32.86, 32.76 and 19.98 for fat weight accounting for LW, fat area at LV5 accounting for LW, fat area at TV8 accounting for LW and fat density at TV8 accounting for LW, respectively (Table 3). An example of the genome-wide RHM Manhattan plot for fat weight accounting for LW is in Fig. 5. The other genome-wide traits RHM Manhattan plots for fat traits are in Additional file 7: Figures S18, S19 and S20, whereas for those that reached the suggestive threshold are in Additional file 8: Figures S21, S22 and S23.

**Table 3:** Regional heritability (h2w) for fat traits, for windows of the genome that were significant at both the genome-wide (p < 0.05) and suggestive levels

Trait	Chr	Windows	Start_position	End_position	LRT^a^	H2w
Fat weight accounting for live weight	6	11	27872427	33467474	17.52	0.07
	6	12	30301209	36839547	35.58	0.07
	6	13	33493183	39305012	37.78	0.06
	6	14	36914376	41901628	26.62	0.08
	6	15	39327441	44214759	10.18	0.06
	6	16	41926986	46660059	9.30	0.07
Fat area at ischium accounting for live weight	6	12	30301209	36839547	10.04	0.03
	6	13	33493183	39305012	10.66	0.03
	13	27	75498647	81506987	9.52	0.07
Fat area at 5th lumbar vertebra	3	8	20116222	25175816	9.12	0.07
Fat area at 5th lumbar vertebra accounting for live weight	6	11	27872427	33467474	13.34	0.07
	6	12	30301209	36839547	29.76	0.06
	6	13	33493183	39305012	32.86	0.05
	6	14	36914376	41901628	23.04	0.07
Fat area at 8th thoracic vertebra	3	8	20116222	25175816	11.02	0.08
Fat area at 8th thoracic vertebra accounting for live weight	6	11	27872427	33467474	16.22	0.06
	6	12	30301209	36839547	32.26	0.06
	6	13	33493183	39305012	32.76	0.05
	6	14	36914376	41901628	23.50	0.07
	6	15	39327441	44214759	12.86	0.07
	6	16	41926986	46660059	13.48	0.08
	12	25	65938940	70532044	11.64	0.07
	12	26	68420308	73046697	9.48	0.05
Fat density at ischium	2	63	166140810	171123855	9.26	0.08
Fat density at ischium accounting for live weight	2	60	158192547	163334761	9.60	0.07
Fat density at 8th thoracic vertebra accounting for live weight	6	8	20138686	24984143	9.46	0.10
	6	12	30301209	36839547	17.52	0.07
	6	13	33493183	39305012	18.46	0.07
	6	14	36914376	41901628	19.98	0.10
	6	15	39327441	44214759	18.72	0.13
	6	16	41926986	46660059	13.64	0.13

*Chr* chromosome

^a^The genome-wide LRT threshold was equal to 13.54, corresponding to a p value less than 1.17 × 10^−4^ and the suggestive LRT threshold was equal to 9.27, corresponding to a p value less than 2.33 × 10^−3^

**Fig. 6:**
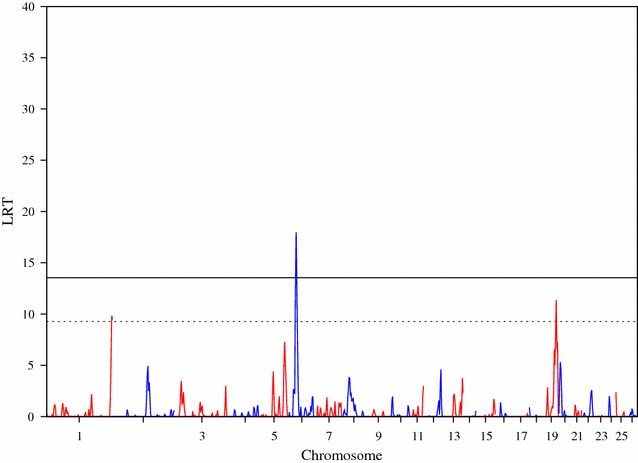
Manhattan plot of the LRT across the genome for muscle density at the 8th thoracic vertebra accounting for live weight, using regional heritability mapping. Genome-wide threshold (p < 0.05) is represented as a *solid line* and suggestive threshold as a *dashed line*

Only one muscle trait (i.e. muscle density at TV8 accounting for LW) was above the genome-wide significance threshold (p < 0.05) associated with a region on OAR6. However, there were nine other traits associated with regions above the suggestive threshold (Table 4). The RHM Manhattan plot for muscle density at TV8 accounting for LW is in Fig. 6. The suggestive Manhattan plots for other muscle traits are in Additional file 8: Figures S24, S25, S26 S27, S28, S29, S30, S31 and S32. Other significant regions were identified on OAR2, 3 and 12.

**Table 4:** Regional heritability (h2w) for muscle traits, for windows of the genome that were significant at both the genome-wide (p < 0.05) and suggestive levels

Trait	Chr	Windows	Start_position	End_position	LRT^a^	H2w
Muscle weight	1	45	123981638	128715957	9.26	0.05
	22	1	259746	6415933	10.22	0.08
	24	8	24040495	31229367	11.98	0.11
	24	9	28078898	34345815	9.84	0.10
Muscle area at ischium	1	45	123981638	128715957	9.50	0.06
Muscle area at 5th lumbar vertebra accounting for live weight	6	18	46782313	51906546	10.62	0.06
Muscle area at 8th thoracic vertebra	16	18	47699158	53439521	11.94	0.15
Muscle area at 8th thoracic vertebra accounting for live weight	16	16	41803341	47620181	10.78	0.09
Muscle density at ischium	26	3	6601755	12382671	10.11	0.10
Muscle density at ischium accounting for live weight	26	3	6601755	12382671	9.58	0.09
Muscle density at 5th lumbar vertebra	22	14	36519601	41316391	10.52	0.05
	22	15	38924538	44247327	9.82	0.05
Muscle density at 8th thoracic vertebra	1	98	272509749	275593974	12.00	0.06
Muscle density at 8th thoracic vertebra accounting for live weight	1	98	272509749	275593974	9.83	0.06
	6	12	30301209	36839547	12.02	0.06
	6	13	33493183	39305012	17.97	0.09
	6	14	36914376	41901628	13.19	0.07
	19	18	51172866	56956148	11.37	0.09

*Chr* chromosome

^a^The genome-wide LRT threshold was equal to 13.54, corresponding to a p value less than 1.17 × 10^−4^ and the suggestive LRT threshold was equal to 9.27, corresponding to a p value less than 2.33 × 10^−3^

For other traits, such as bone proportion, total carcass weight, muscle to bone ratio and LW, only regions reaching the suggestive threshold were identified (Table 5). The corresponding Manhattan plots are in Additional file 8: Figures S33, S34, S35 and S36.

**Table 5:** Regional heritability (h2w) for proportion traits, for windows of the genome that were significant at both the genome-wide (p < 0.05) and suggestive levels

Trait	Chr	Windows	Start_position	End_position	LRT^a^	H2w
Bone percentage	15	20	56988645	61846859	13.02	0.10
	15	21	59267493	64256466	9.70	0.08
	22	1	259746	6415933	9.50	0.10
	22	13	34060763	38910604	9.90	0.07
	22	14	36519601	41316391	10.28	0.06
	22	15	38924538	44247327	10.34	0.06
Total carcass weight	22	1	259746	6415933	10.28	0.07
	24	8	24040495	31229367	12.10	0.11
Muscle to bone ratio	15	20	56988645	61846859	11.66	0.08
	15	21	59267493	64256466	10.89	0.09
Live weight	22	1	259746	6415933	9.88	0.08
	24	8	24040495	31229367	10.72	0.10

*Chr* chromosome

^a^The genome-wide LRT threshold was equal to 13.54, corresponding to a p value less than 1.17 × 10^−4^ and the suggestive LRT threshold was equal to 9.27, corresponding to a p value less than 2.33 × 10^−3^

**Fig. 7:**
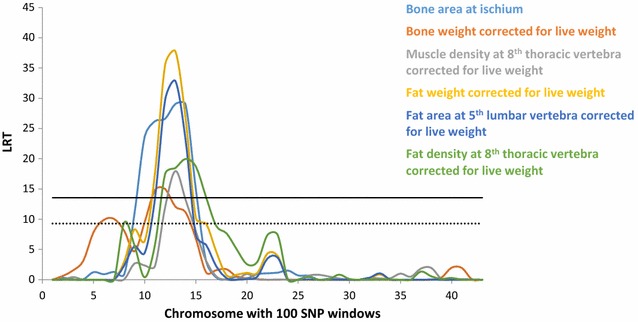
Manhattan plot of the LRT across OAR6 for bone, fat and muscle traits, using regional heritability mapping. Genome-wide threshold (p < 0.05) is represented as a *solid line* in *black* and suggestive threshold as a *dashed line* in *black*

A summary of the regions that were identified on OAR6 for bone, fat and muscle traits and met the genome-wide significance threshold (p < 0.05) using the regional RHM approach is in Fig. 7. Linkage disequilibrium was high within the 2-Mb regions that flanked the most significant SNP in our study (OAR6_40855809); LD results for r^2^, D’ and the identified four haplotype blocks are in Additional file 9: Figures S37, S38 and S39.

## Discussion

Heritabilities for carcass composition traits that were measured by CT, had been previously estimated using the same population and trait data and pedigree relationship matrices only [16]. In the current study, we confirmed these previous heritability estimates (moderate to high) by exploiting SNP genotype data and kinship relationship matrices. The pedigree-based estimates tended to be higher than those calculated here, which may be explained by the data structure, i.e. low LD between SNPs and causal variants among other reasons (for a detailed review on missing heritability, see [28]). Previously, high genetic correlations between carcass composition traits that were measured by CT (hereafter referred to as CT traits) and meat quality for this population were reported [16]. Such high correlations should, in principle, lead to correlated responses for the more expensive to measure traits when selecting for one trait. Other studies reported moderate heritabilities (0.25 to 0.36) for CT traits including carcass fat, carcass muscle and bone, and moderate to high genetic correlations with weaning weight [17]. However, these authors found low genetic correlations between ultrasound muscle depth and CT traits.

The most striking result in our study was the association between a region that spanned ~2 Mb on OAR6 and several carcass composition traits that are arguably related. Although the QTL plots across OAR6 for different traits (Fig. 7) show some overlaps between traits, it is not possible at this stage, to know if we are dealing with a single gene with pleiotropic effects or multiple genes with different effects. Addressing this question through genetic analyses requires access to recombination events which would allow dissection of the candidate region.

The region on OAR6 between 35 and 38 Mb has also been shown to have effects on body weight both in the Scottish Blackface population analyzed here [29] and in Australian Merino sheep [30], and on other meat quality traits in a multi-breed sheep population [31]. The proportions of genetic variance explained by the SNPs were larger for bone area at ISC (44 to 55 %) in our study than for body weight (7.22 %) in the Australian Merino sheep study. However, allele substitution effects were larger (1.54 to 2.34 kg) in the Australian study than in our study on Blackface sheep (0.92 to 1.41 kg) [See Additional file 4: Table S6]. This is probably due to the differences in body size between Australian Merino and Scottish Blackface sheep.

In a study on the detection of selection signals between sheep breeds using global *F*_ST_, Kijas et al. [32] identified the same region on OAR6 as in our study among the 31 regions that they found. However, in their study, the strongest signal was found on OAR10 which carries the *RXFP2* gene that these authors suggested as involved in bone mass [33]. Moreover, since only the loci with major effects, which are private to one or two breeds such as the *myostatin* mutation in Texel sheep, were not detected in their between-breed analyses, we can infer that the allelic effects due to loci on OAR6 are common to more than two breeds. Another study on signatures of selections using the Sheep HapMap dataset (including 71 breeds) and statistical methods that took the hierarchical structure of sheep populations into account, exploited LD information and focused on the age of the selection signatures to identify a region on OAR6 that contains two genes, *ABCG2* and *NCAPG* which are within our region of interest [34]. The selection signature in this region of OAR6 was detected for Central European, Italian and South West European sheep breeds, but they did not include Blackface sheep. Using an *F*_ST_ approach, Gutierrez-Gil et al. [35] compared five sheep dairy breeds with five non-dairy breeds from the Sheep HapMap data and identified a selection signature on OAR6 that encompasses the *ABCG2*/*SPP1* genes in the region of interest. In a different study using Chinese sheep breeds, the same region on OAR6 was identified using population mapping techniques [36]. Finally, Xu et al. [37], based on the detection of selective sweeps identified a region on the bovine genome that contains the same candidate genes (*LCORL* and *NCAPG*) that we found in our region of interest.

We examined a 2-Mb region on OAR6 between 35.34 and 37.40 Mb that flanked our most significant SNP in the sheep reference genome (Ovis_aries_v3.1 [38]) as annotated by Ensembl (http://www.ensembl.org). We identified several positional candidate genes with functions that are relevant to the traits of interest i.e., *OST/SPP1*, *MEPE*, *IBSP*, *LCORL* and *NCAPG*.

The *OST/SPP1* gene, which encodes the secreted phosphoprotein 1, also known as osteopontin, maps to OAR6 between 36,651,734 and 36,658,288 bp and plays a role in bone formation in different species including humans and mice [39–41]. The *MEPE* (*matrix extracellular phosphoglycoprotein*) gene is also known to play a role in bone-related traits in several species including humans, mice and cattle [42–44]. The *IBSP* gene encodes integrin-binding sialoprotein, which is a major structural component of the bone matrix; it is involved in bone diseases [39] and affects skeletal development in mice [45, 46].

Several studies have reported SNPs in regions near the *NCAPG* and *LCORL* genes that are involved in growth and body size, for example in body composition and meat quality traits in chicken [47], calf size at birth and adult stature in cattle [48], withers height and body size in horses [49–51], human height [52, 53] and selective sweeps for growth in pig [54]. An association between a SNP in the *NCAPG*-*LCORL* locus and feed intake, average daily gain, meat and carcass traits has also been found for beef cattle [55]. Lindholm-Perry et al. [56] showed that *NCAPG* and LCORL are expressed in bovine adipose and muscle tissues, these genes being located in a chromosomal region that is associated with feed intake and average daily gain in cattle. *NCAPG* was also reported to be associated with body size in cattle [57, 58].

In addition to the striking effects of the region of interest on OAR6 on CT traits, both the GWAS and RHM analyses detected QTL on other chromosomes, which are much less corroborated by other studies. For example, although Cavanagh et al. [6] reported QTL on OAR1 with effects on carcass bone, carcass lean and lean percentage in carcass in Australian Merino sheep, we did not detect any QTL on OAR1 for these traits in the Scottish Blackface population.

However, we identified SNPs at the telomeric end of OAR1 that were associated ($p = 7.28 \times 10^{ - 7}$) with muscle density at TV8 (Fig. 3). McRae et al. [59] detected a QTL for live weight on OAR1 in a Charollais commercial sheep population and the same QTL was later confirmed in independent populations of commercial Charollais and Suffolk sheep [60]. Several QTL for lamb flavor, weight at slaughter, bone density at ISC, hot carcass weight and meat colour were mapped to OAR1 [16]. Karamichou et al. [16] also noted that muscle density was the CT trait that was most consistently related to meat quality traits and had moderate to strongly negative genetic correlations with live weight, fat class, subcutaneous fat score, dry matter proportion, juiciness, flavor and overall liking. Therefore, it may be interesting to use muscle density in selection programs to improve meat quality traits.

We found two other genome-wide significant QTL for bone density at TV8 accounting for LW on OAR3 and bone density at LV5 on OAR24 (Table 2). Raadsma et al. [61] reported QTL with sex-specific effects for body weight and growth rate in the same region on OAR24 using microsatellite markers. Based on a sheep GWAS, Zhang et al. [62] identified SNPs that reached genome-wide significance for post-weaning gain and were located in about the same region as our region of interest on OAR3 and also for shin circumference on OAR24 but in a different region to that found here. Regions of selective sweeps were mapped to OAR3 in various studies [32, 34–36] but none were within our region of interest on OAR3 except that described in [35].

In the region on OAR3 (between 63,958,505 and 75,607,095 bp) that we detected by RHM for bone proportion, we identified several genes which may be related to bone traits. Among these: (1) *EFEMP1*, although not well annotated, has been shown in in situ analyses to be expressed in the condensing mesenchyme, which gives rise to bone and cartilage, and in developing bone structures of the cranial and axial skeleton during murine embryogenesis [63]; (2) *SPTBN1*, which is involved in healing properties of human bones [64] and is associated with human osteoporosis [65, 66]; and (3) *FSHR*, which influences bone remodeling and osteoclast proliferation activity in postmenopausal women [67].

OAR24 also harbored several important candidate genes, i.e.: (1) *SH2B1* was identified in Japanese women as one of the determining loci for bone mass, especially after menopause [68]; (2) *MAPK3* is involved in post-menopausal osteoporosis in women [69]; (3) *TBX6*, for which Abe et al. [70] suggested that its partial dysfunction led to congenital vertebral malformations in both humans and rats; furthermore, Sparrow et al. [71] reported that a mutation in this gene was the likely cause of spondylocostal dysostosis in some human families [71]; (4) *KIF22* for which mutations have been linked to bone diseases in humans [72, 73]; (5) *IL4R* is a candidate gene because of its known role in cartilage homeostasis and osteoarthritis in humans [74–77]; and (6) *IL21R*, which may have biological functions that are relevant to bone metabolism in humans [78–80].

On OAR1, the only candidate gene that we could identify was *KAT2B* which is involved in cell growth and differentiation.

We analysed our data with two different detection approaches (i.e. single SNP association versus RHM) that both identified many relevant chromosomal regions. However, RHM captured more regions than single SNP association, which is consistent with a previous study on nematode resistance [29]. Previously, Nagamine et al. [24] showed that RHM performs better than a standard association analysis, especially when SNPs do not have large effects, because it integrates the variance that is contributed by both rare and common variants into a single estimate of the additive variance, which potentially allows the identification of loci that cannot be found by single SNP association.

## Conclusions

We identified regions on OAR1, 3, 24 and particularly on OAR6 that are associated with muscle, fat and bone traits. Since there is evidence that these CT traits are genetically correlated with meat quality traits, the associated SNPs have potential applications in selective breeding for improved meat quality. Further studies are required to determine whether the associations between the region on OAR6 and several carcass traits are caused by a single gene with multiple effects or multiple closely-linked genes. Unfortunately, due to the high LD observed in this region, high-resolution genetic analysis will be difficult.

## Additional files

10.1186/s12711-016-0191-3 Heritability estimates for CT traits using kinship and pedigree-based matrices. Tables for estimates of heritability and variance components using both kinship and pedigree-based relation matrices for bone, muscle, fat, and proportion traits.
10.1186/s12711-016-0191-3 Genome-wide Manhattan plot for bone weight using GenABEL software.
10.1186/s12711-016-0191-3 QQ-plots for bone proportion, bone area at the ischium muscle, bone weight, fat area at TV8 accounting for live weight, fat weight accounting for live weight, proportion of killing out percentage and muscle density at TV8, obtained by using the GenABEL software.
10.1186/s12711-016-0191-3 Estimates of SNP effect size and allele frequencies for the four top SNPs. Tables with the summary statistics for minor allele frequencies, allele substitution effects, dominance effects, proportions of genetic and phenotypic variances explained by top four SNPs, and proportions of both genetic and phenotypic deviations across the CT traits of bone, muscle, fat and proportion traits.
10.1186/s12711-016-0191-3 Genome-wide Manhattan plot for bone weight accounting for live weight (LW), bone area at ischium accounting for LW, bone density at LV5, and bone density at TV8 accounting for LW using regional heritability mapping.
10.1186/s12711-016-0191-3 Suggestive Manhattan plots for bone traits using regional heritability mapping.
10.1186/s12711-016-0191-3 Genome-wide Manhattan plots for fat traits using regional heritability mapping.
10.1186/s12711-016-0191-3 Suggestive Manhattan plots for fat, muscle and proportion traits using regional heritability mapping.
10.1186/s12711-016-0191-3 Linkage disequilibrium (r^2^ or D′ values) and phased population haplotypes within the region between 35,347,156 and 37,533,664 bp on OAR6.
