# Supplementary material for: Genome-wide association reveals QTL for growth, bone and in vivo carcass traits as assessed by computed tomography in Scottish Blackface lambs
Source: Genet Sel Evol. 2016 Feb 8;48:11. doi: 10.1186/s12711-016-0191-3 (PMC4745175; doi:10.1186/s12711-016-0191-3)
Supplement: Supplementary file 1 — 10.1186/s12711-016-0191-3 Heritability estimates for CT traits using kinship and pedigree-based matrices. Tables for estimates of heritability and variance components using both kinship and pedigree-based relation matrices for bone, muscle, fat, and proportion traits. [file 12711_2016_191_MOESM1_ESM.docx]

**Additional file 1**

**Table S1 Heritability estimates for bone traits using both the kinship and the pedigree-based relationship matrices**

|  | **Bone weight** | **Bone weight with live weight** | **Bone area at ischium** | **Bone area at ischium with live weight** | **Bone area at LV5 with live weight** | **Bone density at ischium** | **Bone density at ischium with live weight** | **Bone density at LV5** | **Bone density at TV8a** |
| --- | --- | --- | --- | --- | --- | --- | --- | --- | --- |
| **Kinship** |  |  |  |  |  |  |  |  |  |
| σ^2^_a_ | 17555 | 2603 | 10569 | 8153 | 1348 | 203 | 189 | 571 | 93 |
| σ^2^_p_ | 61675 | 9193 | 71208 | 58804 | 6960 | 701 | 621 | 955 | 445 |
| se | 4269 | 628 | 4640 | 3787 | 457 | 48 | 43 | 72 | 30 |
| h^2^ | 0.29 | 0.28 | 0.15 | 0.14 | 0.19 | 0.29 | 0.30 | 0.60 | 0.21 |
| se | 0.10 | 0.09 | 0.09 | 0.09 | 0.09 | 0.10 | 0.10 | 0.09 | 0.10 |
| **Pedigree** |  |  |  |  |  |  |  |  |  |
| σ^2^_a_ | 34240 | 3023 | 10057 | 8685 | 1806 | 234 | 192 | 677 | 125 |
| σ^2^_p_ | 67212 | 9348 | 71308 | 59013 | 7109 | 717 | 628 | 1002 | 456 |
| se | 5621 | 688 | 4781 | 3902 | 506 | 55 | 47 | 84 | 34 |
| h^2^ | 0.51 | 0.32 | 0.14 | 0.15 | 0.25 | 0.33 | 0.31 | 0.68 | 0.27 |
| se | 0.14 | 0.12 | 0.11 | 0.10 | 0.12 | 0.13 | 0.13 | 0.12 | 0.14 |

**Table S2 Heritability estimates for muscle traits using both the kinship and the pedigree-based relationship matrices**

|  | **Muscle weight** | **Muscle area at ischium** | **Muscle area at LV5 with live weight** | **Muscle area at TV8** | **Muscle area at TV8 with live weight** | **Muscle density at ischium** | **Muscle density at ischium with live weight** | **Muscle density at LV5** | **Muscle density at TV8** |
| --- | --- | --- | --- | --- | --- | --- | --- | --- | --- |
| **Kinship** |  |  |  |  |  |  |  |  |  |
| σ^2^_a_ | 341134 | 1258620 | 86007 | 539473 | 166771 | 1.71 | 1.86 | 3.15 | 4.33 |
| σ^2^_p_ | 993020 | 3994700 | 259960 | 1484900 | 623000 | 3.05 | 3.04 | 7.30 | 7.87 |
| se | 69854 | 279290 | 18877 | 104890 | 43646 | 0.24 | 0.24 | 0.53 | 0.59 |
| h^2^ | 0.34 | 0.32 | 0.33 | 0.36 | 0.27 | 0.56 | 0.61 | 0.43 | 0.55 |
| se | 0.10 | 0.10 | 0.11 | 0.10 | 0.11 | 0.10 | 0.09 | 0.10 | 0.09 |
| **Pedigree** |  |  |  |  |  |  |  |  |  |
| σ^2^_a_ | 678601 | 2137050 | 112557 | 951372 | 267137 | 1.43 | 1.54 | 4.16 | 4.69 |
| σ^2^_p_ | 1106200 | 4299900 | 269820 | 1624600 | 655100 | 3.01 | 2.98 | 7.63 | 8.08 |
| se | 93914 | 355420 | 22423 | 136710 | 53239 | 0.25 | 0.25 | 0.62 | 0.67 |
| h^2^ | 0.61 | 0.50 | 0.42 | 0.59 | 0.41 | 0.48 | 0.52 | 0.54 | 0.58 |
| se | 0.13 | 0.14 | 0.15 | 0.13 | 0.14 | 0.14 | 0.14 | 0.13 | 0.13 |

**Table S3 Heritability estimates for fat traits using both the kinship and the pedigree-based relationship matrices**

| **Trait** | **Fat weight with live weight** | **Fat area at ischium with live weight** | **Fat area at LV5** | **Fat area at LV5 with live weight** | **Fat area at TV8** | **Fat area at TV8 with live weight** | **Fat density at ischium** | **Fat density at ischium with live weight** |
| --- | --- | --- | --- | --- | --- | --- | --- | --- |
| **Kinship** |  |  |  |  |  |  |  |  |
| σ^2^_a_ | 191479 | 453438 | 416280 | 279057 | 1657240 | 1086460 | 4.57 | 3.22 |
| σ^2^_p_ | 247540 | 765890 | 705750 | 397380 | 3067500 | 1435300 | 20.48 | 16.21 |
| se | 19192 | 58797 | 53208 | 30572 | 228390 | 111690 | 1.40 | 1.10 |
| h^2^ | 0.77 | 0.59 | 0.59 | 0.70 | 0.54 | 0.76 | 0.22 | 0.20 |
| se | 0.07 | 0.09 | 0.09 | 0.08 | 0.09 | 0.07 | 0.10 | 0.10 |
| **Pedigree** |  |  |  |  |  |  |  |  |
| σ^2^_a_ | 270937 | 378255 | 568818 | 391737 | 2530980 | 1522830 | 4.80 | 0.36 |
| σ^2^_p_ | 280370 | 755080 | 761910 | 442750 | 3364500 | 1616100 | 20.70 | 15.60 |
| se | 24217 | 61347 | 63640 | 38744 | 283920 | 140390 | 1.54 | 0.99 |
| h^2^ | 0.97 | 0.50 | 0.75 | 0.88 | 0.75 | 0.94 | 0.23 | 0.02 |
| se | 0.09 | 0.13 | 0.11 | 0.10 | 0.11 | 0.10 | 0.14 | 0.09 |

**Table S4 Heritability estimates for proportion traits using both the kinship and the pedigree-based relationship matrices**

|  | **Bone proportion** | **Total carcass weight** | **Muscle to bone ratio** | **Live weight** |
| --- | --- | --- | --- | --- |
| **Kinship** |  |  |  |  |
| σ^2^_a_ | 0.00019 | 1547260 | 0.02 | 6.19 |
| σ^2^_p_ | 0.00039 | 4162700 | 0.04 | 19.19 |
| se | 0.00003 | 295390 | 0.00 | 1.34 |
| h^2^ | 0.47 | 0.37 | 0.43 | 0.32 |
| se | 0.10 | 0.10 | 0.10 | 0.10 |
| **Pedigree** |  |  |  |  |
| σ^2^_a_ | 0.00030 | 2947180 | 0.03 | 11.61 |
| σ^2^_p_ | 0.00043 | 4624800 | 0.04 | 21.03 |
| se | 0.00004 | 389400 | 0.00 | 1.75 |
| h^2^ | 0.70 | 0.64 | 0.63 | 0.55 |
| se | 0.12 | 0.12 | 0.12 | 0.13 |
