# Supplementary material for: Genome-wide association reveals QTL for growth, bone and in vivo carcass traits as assessed by computed tomography in Scottish Blackface lambs
Source: Genet Sel Evol. 2016 Feb 8;48:11. doi: 10.1186/s12711-016-0191-3 (PMC4745175; doi:10.1186/s12711-016-0191-3)
Supplement: Supplementary file 2 — 10.1186/s12711-016-0191-3 Genome-wide Manhattan plot for bone weight using GenABEL software. [file 12711_2016_191_MOESM2_ESM.docx]

**Additional file 2**

**Figure S1 Manhattan plot for bone weight using GenABEL software**
