# Supplementary material for: Genome-wide association reveals QTL for growth, bone and in vivo carcass traits as assessed by computed tomography in Scottish Blackface lambs
Source: Genet Sel Evol. 2016 Feb 8;48:11. doi: 10.1186/s12711-016-0191-3 (PMC4745175; doi:10.1186/s12711-016-0191-3)
Supplement: Supplementary file 3 — 10.1186/s12711-016-0191-3 QQ-plots for bone proportion, bone area at the ischium muscle, bone weight, fat area at TV8 accounting for live weight, fat weight accounting for live weight, proportion of killing out percentage and muscle density at TV8, obtained by using the GenABEL software. [file 12711_2016_191_MOESM3_ESM.docx]

**Additional file 3**

**Figure S2 QQ plot for bone proportion (mmb_P) using GenABEL software**

**Figure S3 QQ plot for bone area at the ischium using GenABEL software**

**Figure S4 QQ plot for bone weight using GenABEL software**

**Figure S5 QQ plot for fat area at 8^th^ thoracic vertebra accounting for live weight using GenABEL software**

**Figure S6 QQ plot for fat weight accounting for live weight using GenABEL software**

**Figure S7 QQ plot for proportion of killing out percentage (mmKO_P) using GenABEL software**

**Figure S8 QQ plot for muscle density at 8^th^ thoracic vertebra using GenABEL software**
