# Supplementary material for: Genome-wide association reveals QTL for growth, bone and in vivo carcass traits as assessed by computed tomography in Scottish Blackface lambs
Source: Genet Sel Evol. 2016 Feb 8;48:11. doi: 10.1186/s12711-016-0191-3 (PMC4745175; doi:10.1186/s12711-016-0191-3)
Supplement: Supplementary file 4 — 10.1186/s12711-016-0191-3 Estimates of SNP effect size and allele frequencies for the four top SNPs. Tables with the summary statistics for minor allele frequencies, allele substitution effects, dominance effects, proportions of genetic and phenotypic variances explained by top four SNPs, and proportions of both genetic and phenotypic deviations across the CT traits of bone, muscle, fat and proportion traits. [file 12711_2016_191_MOESM4_ESM.docx]

**Additional file 4**

**Table S5 P-values and allele frequencies for four various top CT-traits obtained in the ASreml analyses fitting SNP as a fixed effect**

| Trait/SNPname | SNP1 | SNP2 | SNP3 | SNP4 |
| --- | --- | --- | --- | --- |
| Chromosome | 6 | 6 | 6 | 1 |
| fat_wt (g) | NS | 0.319 | 0.705 | 0.043 |
| mus_wt (g) | <.001 | 0.007 | 0.003 | 0.014 |
| bon_wt (g) | <.001 | <.001 | <.001 | 0.018 |
| cs_tot_wt (g) | 0.006 | 0.301 | 0.16 | 0.017 |
| LW (kg) | <.001 | 0.005 | 0.002 | 0.013 |
| KO_P | 0.003 | <.001 | <.001 | 0.438 |
| f_P | NS | 0.006 | 0.041 | 0.037 |
| m_P | NS | 0.005 | 0.023 | 0.081 |
| b_P | NS | 0.104 | 0.158 | 0.074 |
| M:B | NS | 0.404 | 0.527 | 0.185 |
| fat_area_ISC (mm^2^) | 0.48 | 0.312 | 0.819 | 0.084 |
| fat_density_ISC | <.001 | 0.006 | 0.023 | 0.237 |
| mus_area_ISC (mm^2^) | <.001 | 0.03 | 0.005 | 0.006 |
| mus_density_ISC | 0.093 | 0.854 | 0.269 | 0.16 |
| bon_area_ISC (mm^2^) | <.001 | <.001 | <.001 | 0.114 |
| bon_density_ISC | 0.003 | 0.002 | 0.007 | 0.344 |
| fat_area_ISC_LW (mm^2^) | 0.002 | <.001 | 0.004 | 0.171 |
| fat_area_LV5 (mm^2^) | 0.1 | 0.002 | 0.028 | 0.135 |
| fat_density_LV5 | 0.569 | 0.072 | 0.705 | 0.467 |
| mus_area_LV5 (mm^2^) | <.001 | 0.014 | 0.065 | 0.001 |
| mus_density_LV5 | 0.797 | 0.617 | 0.354 | 0.004 |
| bon_area_LV5 (mm^2^) | <.001 | 0.018 | 0.004 | 0.396 |
| bon_density_LV5 | 0.489 | 0.949 | 0.73 | 0.099 |
| fat_area_LV5_LW (mm^2^) | <.001 | <.001 | <.001 | 0.175 |
| fat_area_TV8 (mm^2^) | 0.165 | 0.012 | 0.055 | 0.106 |
| fat_density_TV8 | 0.059 | <.001 | 0.041 | 0.85 |
| mus_area_TV8 (mm^2^) | 0.008 | 0.15 | 0.15 | 0.047 |
| mus_density_TV8 | 0.066 | 0.196 | 0.133 | <.001 |
| bon_area_TV8 (mm^2^) | <.001 | <.001 | 0.003 | 0.026 |
| bon_density_TV8 | 0.336 | 0.862 | 0.771 | 0.584 |
| fat_area_TV8_LW (mm^2^) | <.001 | <.001 | <.001 | 0.237 |
| Allele Freq p(A) | 0.63 | 0.67 | 0.65 | 0.64 |
| Allele Freq q(G) | 0.37 | 0.33 | 0.35 | 0.36 |

SNP1 = OAR6_40855809

SNP2 = DU178311_404

SNP3 = OAR6_40955920

SNP4 = s66995

wt = weight, mus = muscle, bon = bone, cs_tot_wt = carcass total weight, LW = live weight, KO_P = killing out proportion, f_P = fat proportion, m_P =muscle proportion, b_P = bone proportion, M:b is muscle to bone ratio, ISC = the ischium, LV5 = 5th lumbar vertebrae, TV8 = 8th thoracic vertebrae.

**Table S6 SNP substitution effects (a) for OAR6_40855809 (SNP1), standard errors (SE), dominance effects (dom), proportion of additive genetic variance (Pvargen), proportion of phenotypic variance (PvarPhe), additive genetic standard deviations (SDgen) and phenotypic deviations (SDphen) explained by the SNP**

| Trait | a | SE (a) | p-value (a) | dom | SE (dom) | p-value (dom) | Pvargen | PvarPhe | SDgen | SDphen |
| --- | --- | --- | --- | --- | --- | --- | --- | --- | --- | --- |
| mus_wt (g) | 312.996 | 66.371 | 7.28E-06 | 7.817 | 78.535 | 3.97E-01 | 0.06 | 0.05 | 0.38 | 0.31 |
| bon_wt (g) | 100.195 | 16.547 | 7.79E-09 | -6.801 | 19.652 | 3.76E-01 | 0.12 | 0.08 | 0.49 | 0.40 |
| cs_tot_wt (g) | 426.053 | 134.627 | 2.77E-03 | 29.605 | 159.174 | 3.92E-01 | 0.03 | 0.02 | 0.25 | 0.21 |
| LW (kg) | 1.414 | 0.292 | 3.93E-06 | 0.080 | 0.346 | 3.88E-01 | 0.07 | 0.05 | 0.41 | 0.33 |
| KO_P | -0.402 | 0.123 | 2.10E-03 | 0.049 | 0.145 | 3.77E-01 | 0.02 | 0.02 | -0.22 | -0.21 |
| fat_density_ISC | 0.002 | 0.314 | 3.99E-01 | -1.552 | 0.390 | 1.59E-04 | 0.03 | 0.00 | 0.00 | 0.00 |
| mus_area_ISC (mm^2^) | 598.755 | 136.299 | 3.01E-05 | -6.341 | 162.516 | 3.98E-01 | 0.07 | 0.04 | 0.40 | 0.30 |
| bon_area_ISC | 114.637 | 17.792 | 8.12E-10 | -15.488 | 22.803 | 3.17E-01 | 0.55 | 0.09 | 1.05 | 0.43 |
| bon_density_ISC | -4.682 | 1.878 | 1.80E-02 | 3.701 | 2.292 | 1.08E-01 | 0.07 | 0.02 | -0.33 | -0.18 |
| fat_area_ISC_LW (mm^2^) | -4.927 | 1.873 | 1.28E-02 | 3.710 | 2.248 | 1.02E-01 | 0.04 | 0.02 | -0.25 | -0.18 |
| mus_area_LV5 (mm^2^) | 205.110 | 56.923 | 6.46E-04 | -31.063 | 67.879 | 3.59E-01 | 0.05 | 0.03 | 0.32 | 0.25 |
| bon_area_LV5 (mm^2^) | 25.769 | 6.509 | 1.74E-04 | -3.620 | 7.943 | 3.59E-01 | 0.12 | 0.04 | 0.48 | 0.28 |
| fat_area_LV5_LW (mm^2^) | -5.780 | 1.406 | 9.64E-05 | 0.899 | 1.662 | 3.44E-01 | 0.05 | 0.04 | -0.30 | -0.27 |
| mus_area_TV8 | 262.170 | 83.516 | 3.00E-03 | 84.075 | 99.742 | 2.79E-01 | 0.03 | 0.02 | 0.29 | 0.22 |
| bon_area_TV8 (mm^2^) | 79.076 | 26.866 | 5.39E-03 | -49.609 | 32.544 | 1.25E-01 | 0.07 | 0.03 | 0.32 | 0.21 |
| fat_area_TV8_LW (mm^2^) | -10.664 | 2.722 | 2.04E-04 | 4.794 | 3.206 | 1.30E-01 | 0.04 | 0.04 | -0.27 | -0.25 |

wt = weight, mus = muscle, bon = bone, cs_tot_wt = carcass total weight, LW = live weight, KO_P = killing out proportion, ISC = ischium, LV5 = 5th lumbar vertebrae, TV8 = 8th thoracic vertebrae.

**Table S7 SNP substitution effects (a) for DU178311_404 (SNP2), standard errors (SE), dominance effects (dom), proportion of additive genetic variance (Pvargen), proportion of phenotypic variance (PvarPhe), additive genetic standard deviations (SDgen) and phenotypic deviations (SDphen) explained by the SNP**

| Trait | a | SE (a) | p-value (a) | dom | SE (dom) | p-value (dom) | Pvargen | PvarPhe | SDgen | SDphen |
| --- | --- | --- | --- | --- | --- | --- | --- | --- | --- | --- |
| mus_wt (g) | 195.091 | 73.605 | 1.21E-02 | -51.003 | 84.894 | 3.33E-01 | 0.03 | 0.02 | 0.23 | 0.20 |
| bon_wt (g) | 79.841 | 18.425 | 3.86E-05 | -2.047 | 21.327 | 3.97E-01 | 0.07 | 0.05 | 0.39 | 0.32 |
| LW (kg) | 0.922 | 0.323 | 6.99E-03 | -0.149 | 0.375 | 3.68E-01 | 0.03 | 0.02 | 0.26 | 0.21 |
| KO_P | -0.607 | 0.134 | 1.70E-05 | -0.091 | 0.153 | 3.34E-01 | 0.04 | 0.04 | -0.33 | -0.31 |
| f_P | -0.010 | 0.003 | 4.61E-03 | -0.001 | 0.004 | 3.96E-01 | 0.03 | 0.02 | -0.26 | -0.22 |
| m_P | 0.007 | 3.016 | 4.36E-03 | 0.000 | 0.003 | 3.99E-01 | 0.03 | 0.02 | 0.27 | 0.22 |
| fat_density_ISC | 0.677 | 0.345 | 5.81E-02 | -0.638 | 0.423 | 1.28E-01 | 0.11 | 0.02 | 0.39 | 0.15 |
| bon_area_ISC (mm^2^) | 118.818 | 20.122 | 1.81E-08 | 15.774 | 24.912 | 3.26E-01 | 0.48 | 0.08 | 1.09 | 0.45 |
| bon_density_ISC | -5.429 | 2.047 | 1.20E-02 | 2.569 | 2.468 | 2.32E-01 | 0.09 | 0.03 | -0.38 | -0.21 |
| fat_area_ISC_LW (mm^2^) | -7.786 | 2.035 | 2.89E-04 | 1.183 | 2.397 | 3.53E-01 | 0.08 | 0.04 | -0.40 | -0.29 |
| fat_area_LV5 (mm^2^) | -200.415 | 59.730 | 1.50E-03 | -24.025 | 68.961 | 3.75E-01 | 0.03 | 0.02 | -0.27 | -0.24 |
| fat_area_LV5_LW (mm^2^) | -7.682 | 1.528 | 1.71E-06 | -0.541 | 1.765 | 3.80E-01 | 0.07 | 0.05 | -0.40 | -0.36 |
| fat_density_TV8 | 1.480 | 0.395 | 3.84E-04 | 0.091 | 0.464 | 3.91E-01 | 0.06 | 0.03 | 0.37 | 0.28 |
| bon_area_TV8 (mm^2^) | 95.621 | 29.460 | 2.14E-03 | -23.784 | 35.099 | 3.17E-01 | 0.08 | 0.03 | 0.39 | 0.25 |
| fat_area_TV8_LW (mm^2^) | -14.270 | 2.977 | 5.10E-06 | 1.751 | 3.426 | 3.50E-01 | 0.06 | 0.05 | -0.37 | -0.34 |

wt = weight, mus = muscle, bon = bone, cs_tot_wt = carcass total weight, LW = live weight, KO_P = killing out proportion, f_P = fat proportion, m_P =muscle proportion, ISC = ischium, LV5 = 5th lumbar vertebrae, TV8 = 8th thoracic vertebrae.

**Table S8 SNP substitution effects (a) for OAR6_40955920 (SNP3), standard errors (SE), dominance effects (dom), proportion of additive genetic variance (Pvargen), proportion of phenotypic variance (PvarPhe), additive genetic standard deviations (SDgen) and phenotypic deviations (SDphen) explained by the SNP**

| Trait | a | SE (a) | p-value (a) | dom | SE (dom) | p-value (dom) | Pvargen | PvarPhe | SDgen | SDphen |
| --- | --- | --- | --- | --- | --- | --- | --- | --- | --- | --- |
| mus_wt (g) | 234.580 | 71.208 | 1.84E-03 | 10.964 | 79.937 | 3.95E-01 | 0.04 | 0.02 | 0.24 | 0.28 |
| bon_wt (g) | 83.289 | 17.806 | 8.66E-06 | -6.890 | 20.057 | 3.76E-01 | 0.08 | 0.05 | 0.41 | 0.33 |
| LW (kg) | 1.065 | 0.312 | 1.25E-03 | -0.025 | 0.353 | 3.98E-01 | 0.04 | 0.03 | 0.31 | 0.25 |
| KO_P | -0.481 | 0.131 | 5.11E-04 | 0.096 | 0.145 | 3.20E-01 | 0.04 | 0.03 | -0.26 | -0.25 |
| mus_area_ISC (mm^2^) | 460.812 | 145.576 | 2.76E-03 | 30.614 | 165.362 | 3.92E-01 | 0.04 | 0.02 | 0.31 | 0.23 |
| bon_area_ISC (mm^2^) | 104.451 | 19.587 | 3.77E-07 | -6.832 | 23.488 | 3.82E-01 | 0.44 | 0.07 | 0.96 | 0.39 |
| bon_density_ISC | -4.623 | 1.986 | 2.68E-02 | 3.043 | 2.321 | 1.69E-01 | 0.07 | 0.02 | -0.32 | -0.18 |
| fat_area_ISC_LW (mm^2^) | -5.786 | 1.986 | 5.87E-03 | 1.598 | 2.271 | 3.11E-01 | 0.05 | 0.02 | -0.29 | -0.21 |
| bon_area_LV5 (mm^2^) | 21.972 | 6.944 | 2.77E-03 | -1.243 | 8.077 | 3.94E-01 | 0.08 | 0.03 | 0.41 | 0.24 |
| fat_area_LV5_LW (mm^2^) | -6.549 | 1.493 | 3.10E-05 | 0.683 | 1.667 | 3.67E-01 | 0.06 | 0.04 | -0.34 | -0.30 |
| bon_area_TV8 (mm^2^) | 71.659 | 28.600 | 1.75E-02 | -50.084 | 33.043 | 1.26E-01 | 0.06 | 0.02 | 0.29 | 0.19 |
| fat_area_TV8_LW (mm^2^) | -12.810 | 2.905 | 2.80E-05 | 2.922 | 3.230 | 2.65E-01 | 0.06 | 0.05 | -0.33 | -0.30 |

wt = weight, mus = muscle, bon = bone, cs_tot_wt = carcass total weight, LW = live weight, KO_P = killing out proportion, ISC = ischium, LV5 = 5th lumbar vertebrae, TV8 = 8th thoracic vertebrae.

**Table S9 SNP substitution effects (a) for s66995 (SNP4), standard errors (SE), dominance effects (dom), proportion of additive genetic variance (Pvargen), proportion of phenotypic variance (PvarPhe), additive genetic standard deviations (SDgen) and phenotypic deviations (SDphen) explained by the SNP.**

| Trait | a | SE (a) | p-value (a) | dom | SE (dom) | p-value (dom) | Pvargen | PvarPhe | SDgen | SDphen |
| --- | --- | --- | --- | --- | --- | --- | --- | --- | --- | --- |
| mus_area_ISC (mm^2^) | -155.994 | 142.067 | 2.18E-01 | 460.038 | 169.585 | 1.03E-02 | 0.02 | 0.01 | -0.10 | -0.08 |
| mus_area_LV5 (mm^2^) | -51.961 | 58.924 | 2.70E-01 | 229.801 | 70.189 | 1.96E-03 | 0.02 | 0.01 | -0.08 | -0.06 |
| mus_density_LV5 | 0.698 | 0.194 | 6.48E-04 | 0.151 | 0.231 | 3.22E-01 | 0.05 | 0.03 | 0.34 | 0.26 |
| mus_density_TV8 | 0.974 | 0.199 | 3.22E-06 | 0.227 | 0.237 | 2.52E-01 | 0.09 | 0.05 | 0.46 | 0.35 |

mus = muscle, ISC = ischium, LV5 = 5th lumbar vertebrae, TV8 = 8th thoracic vertebrae.
