# Supplementary material for: Genome-wide association reveals QTL for growth, bone and in vivo carcass traits as assessed by computed tomography in Scottish Blackface lambs
Source: Genet Sel Evol. 2016 Feb 8;48:11. doi: 10.1186/s12711-016-0191-3 (PMC4745175; doi:10.1186/s12711-016-0191-3)

**Additional file 5**

**Figure S9 Manhattan plot for bone weight accounting for live weight using Regional Heritability Mapping**


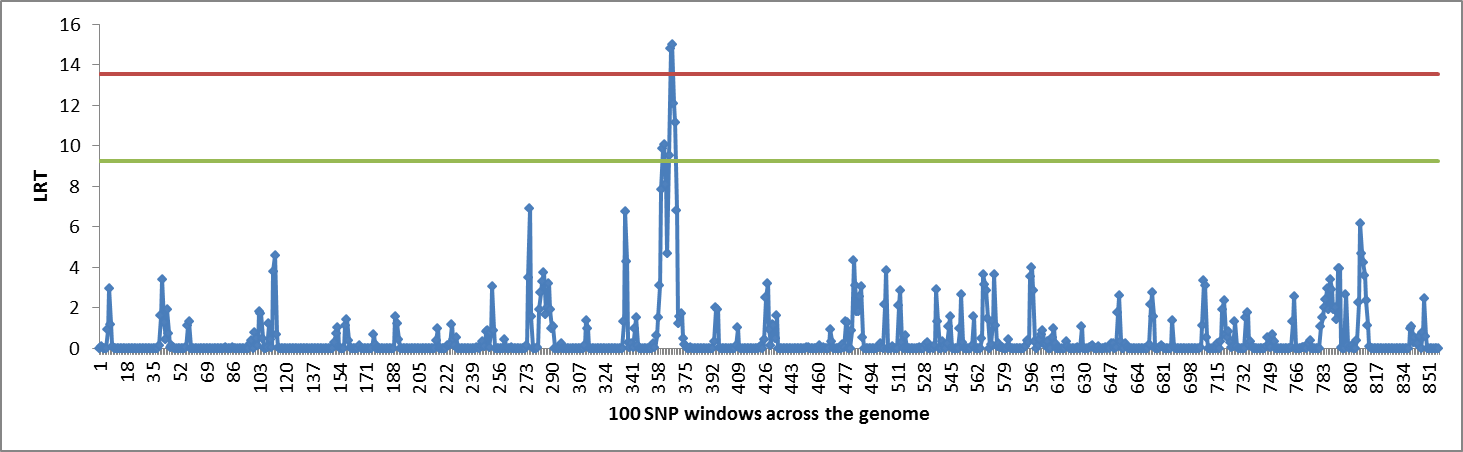


**Figure S10 Manhattan plot for bone area at ischium accounting for live weight using Regional Heritability Mapping**


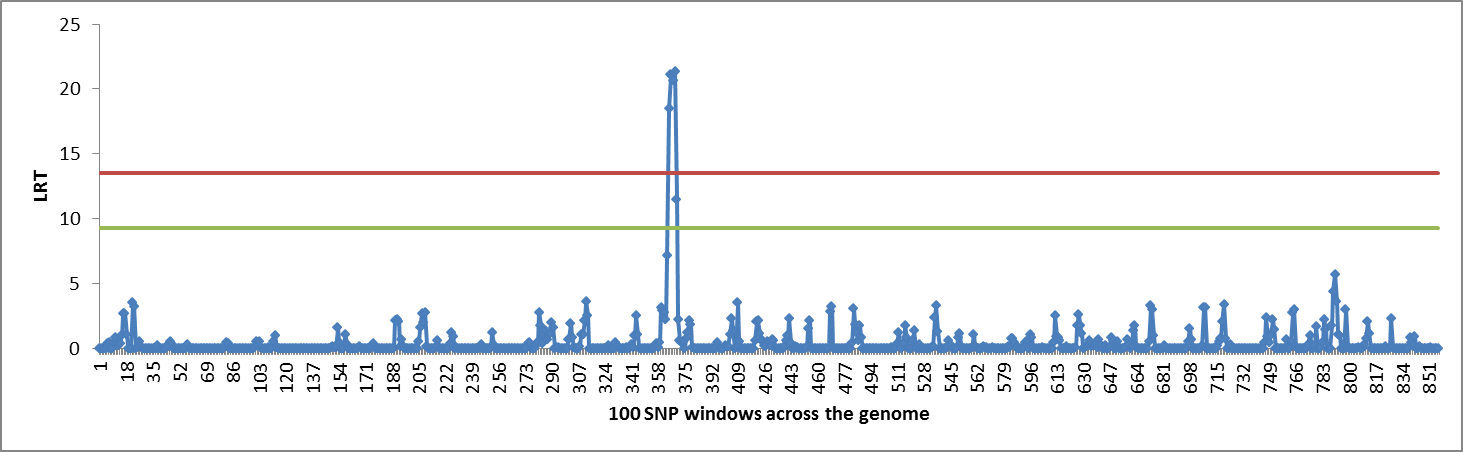


**Figure S11 Manhattan plot for bone density at the 5th lumbar vertebra using Regional Heritability Mapping**


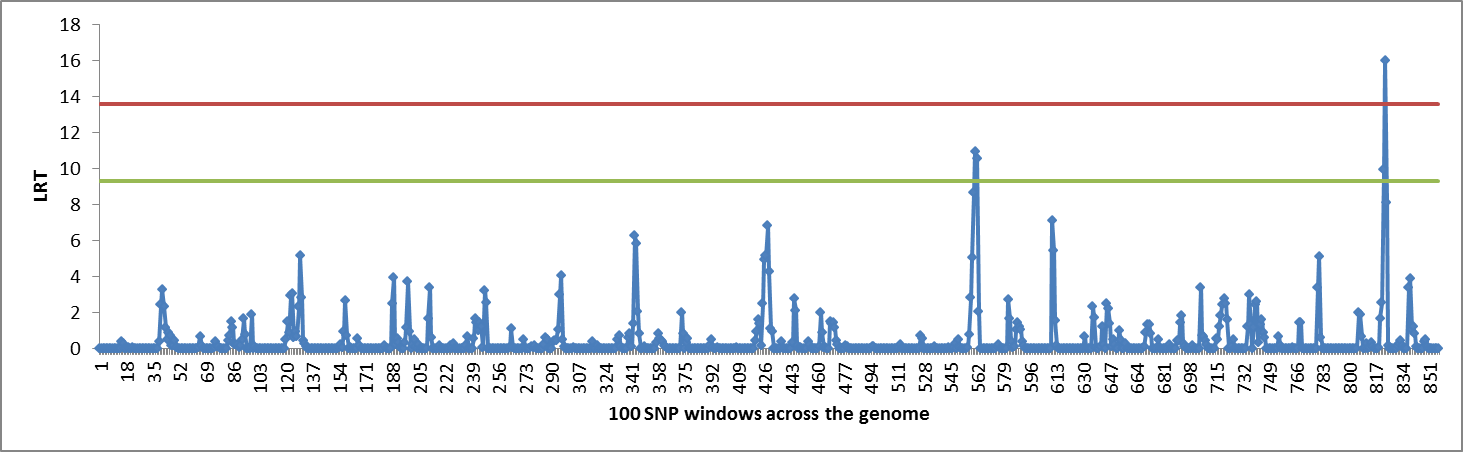


**Figure S12 Manhattan plot for bone density at the 8th thoracic vertebra accounting for weight using Regional Heritability Mapping**


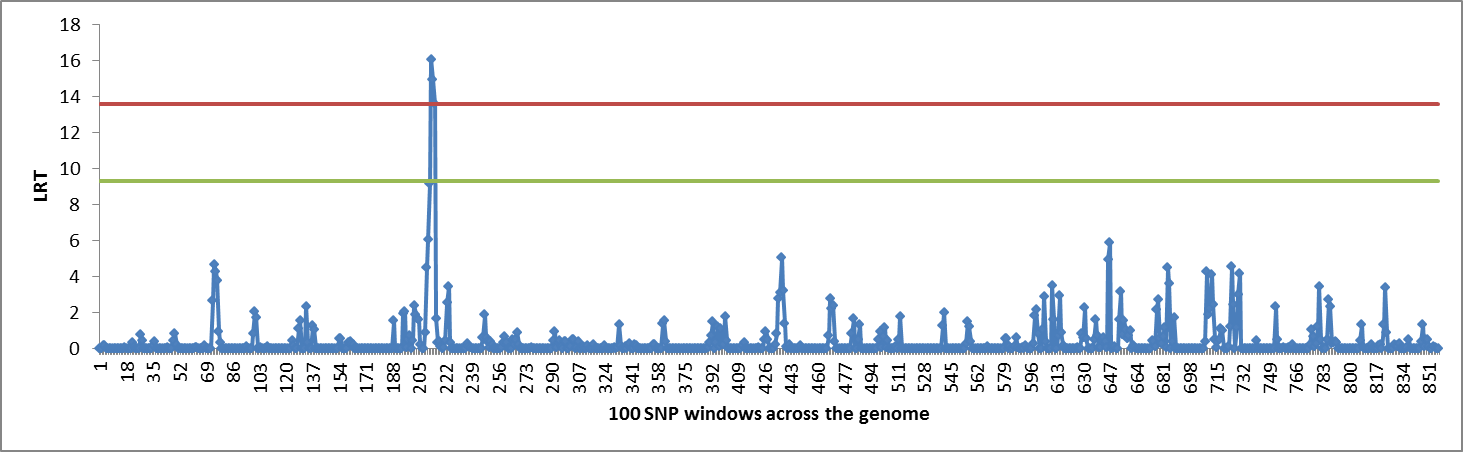

Supplement: Supplementary file 5 — 10.1186/s12711-016-0191-3 Genome-wide Manhattan plot for bone weight accounting for live weight (LW), bone area at ischium accounting for LW, bone density at LV5, and bone density at TV8 accounting for LW using regional heritability mapping. [file 12711_2016_191_MOESM5_ESM.docx]
