# Supplementary material for: Genome-wide association reveals QTL for growth, bone and in vivo carcass traits as assessed by computed tomography in Scottish Blackface lambs
Source: Genet Sel Evol. 2016 Feb 8;48:11. doi: 10.1186/s12711-016-0191-3 (PMC4745175; doi:10.1186/s12711-016-0191-3)

**Additional file 6**

**Figure S13 Manhattan plot for bone weight using Regional Heritability Mapping**


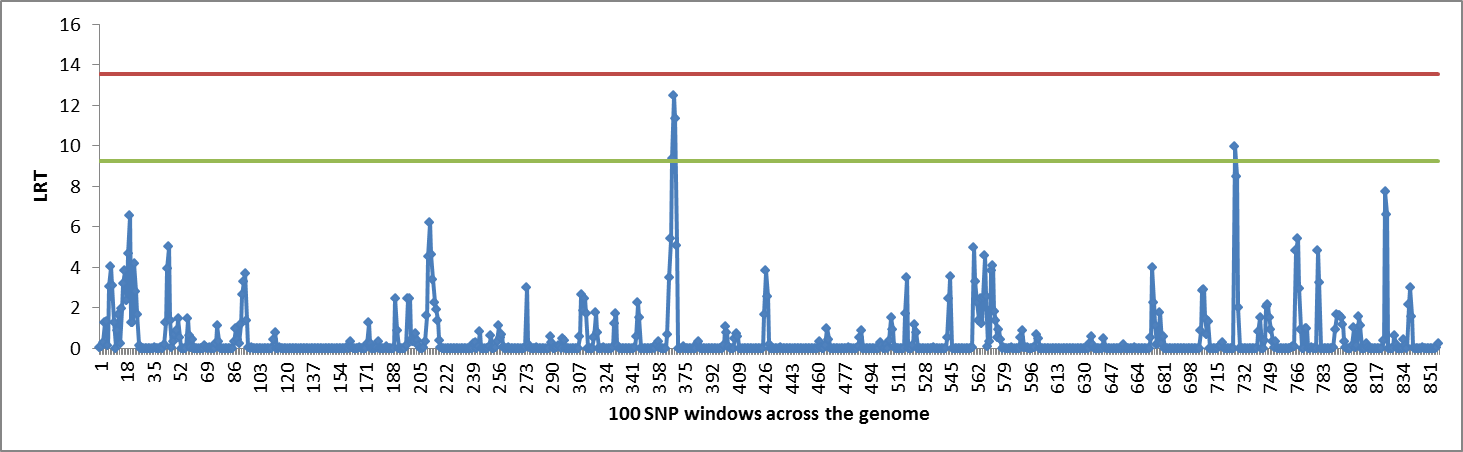


**Figure S14 Manhattan plot for bone area at the 5th lumbar vertebra accounting for live weight using Regional Heritability Mapping**


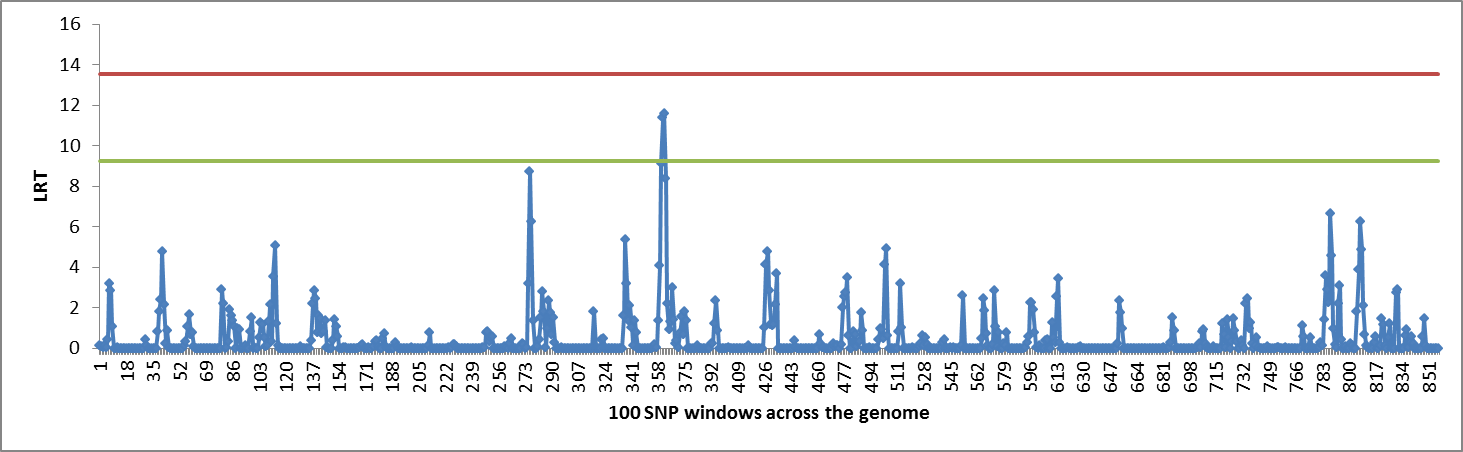


**Figure S15 Manhattan plot for bone density at the ischium using Regional Heritability Mapping**


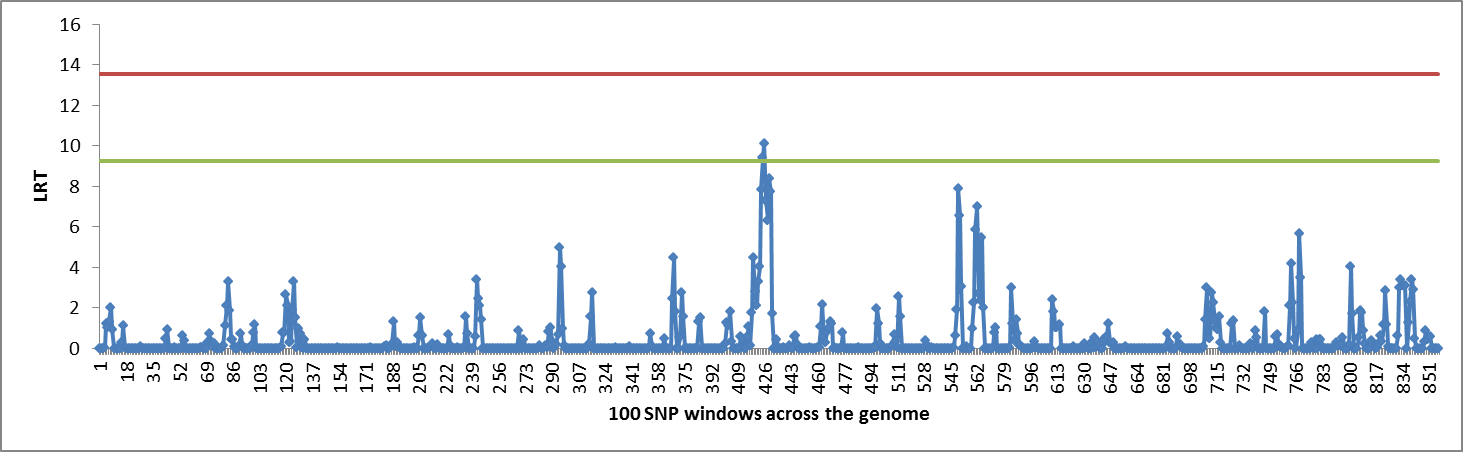


**Figure S16 Manhattan plot for bone density at the ischium accounting for live weight using Regional Heritability Mapping**


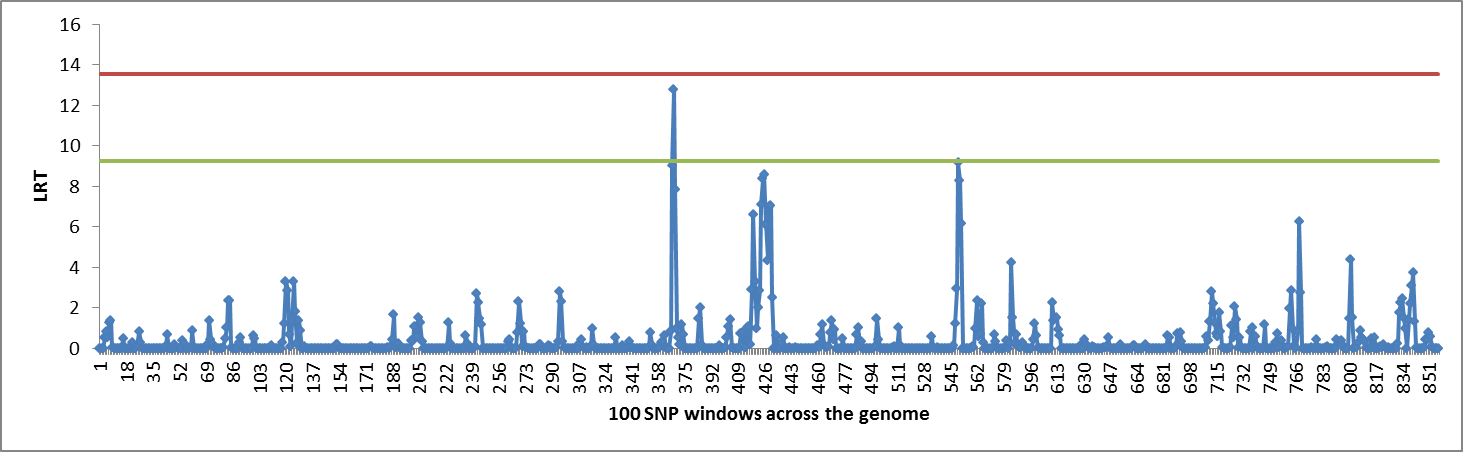


**Figure S17 Manhattan plot for bone density at the 8^th^ thoracic vertebra using Regional Heritability Mapping**


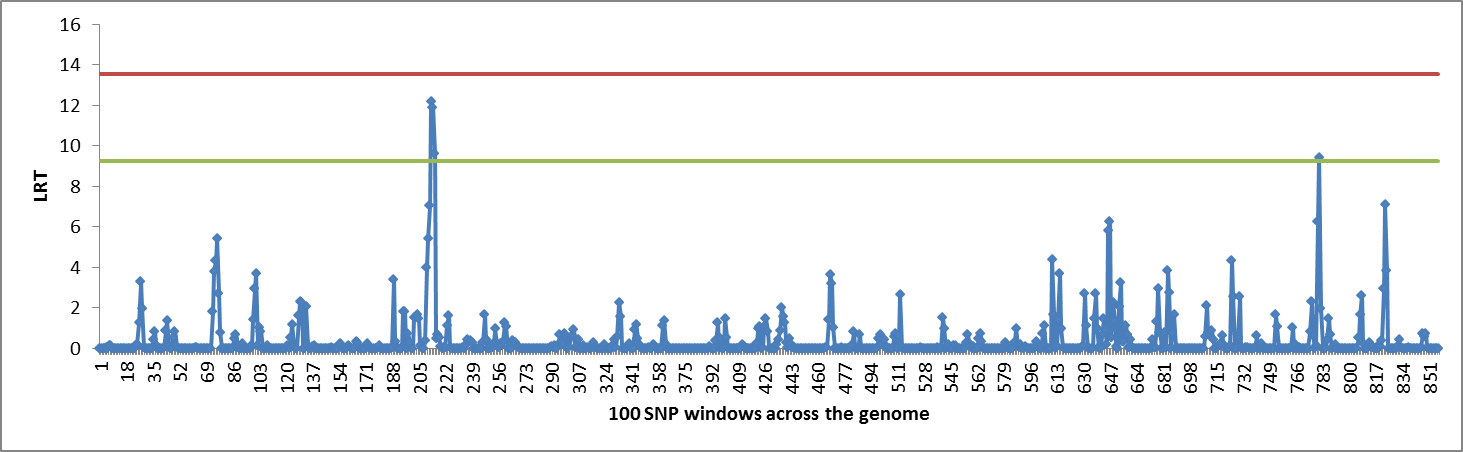

Supplement: Supplementary file 6 — 10.1186/s12711-016-0191-3 Suggestive Manhattan plots for bone traits using regional heritability mapping. [file 12711_2016_191_MOESM6_ESM.docx]
