# Supplementary material for: Genome-wide association reveals QTL for growth, bone and in vivo carcass traits as assessed by computed tomography in Scottish Blackface lambs
Source: Genet Sel Evol. 2016 Feb 8;48:11. doi: 10.1186/s12711-016-0191-3 (PMC4745175; doi:10.1186/s12711-016-0191-3)

**Additional file 7**

**Figure S18 Manhattan plot for fat area at the 5th lumbar vertebra accounting for live weight using Regional Heritability Mapping**


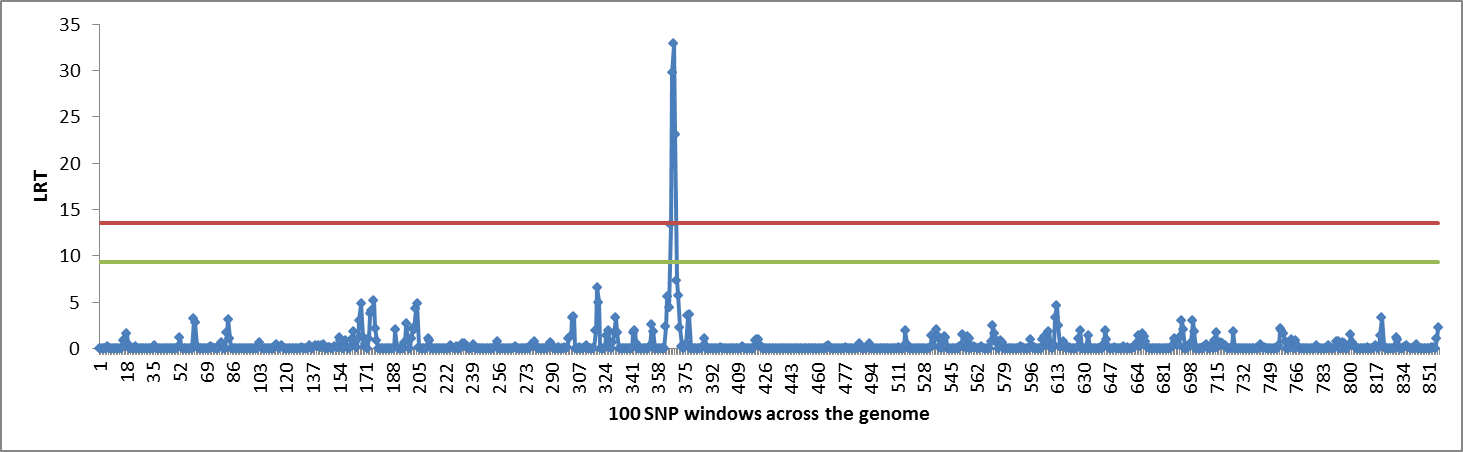


**Figure S19 Manhattan plot for fat area at the 8th thoracic vertebra accounting for live weight using Regional Heritability Mapping**


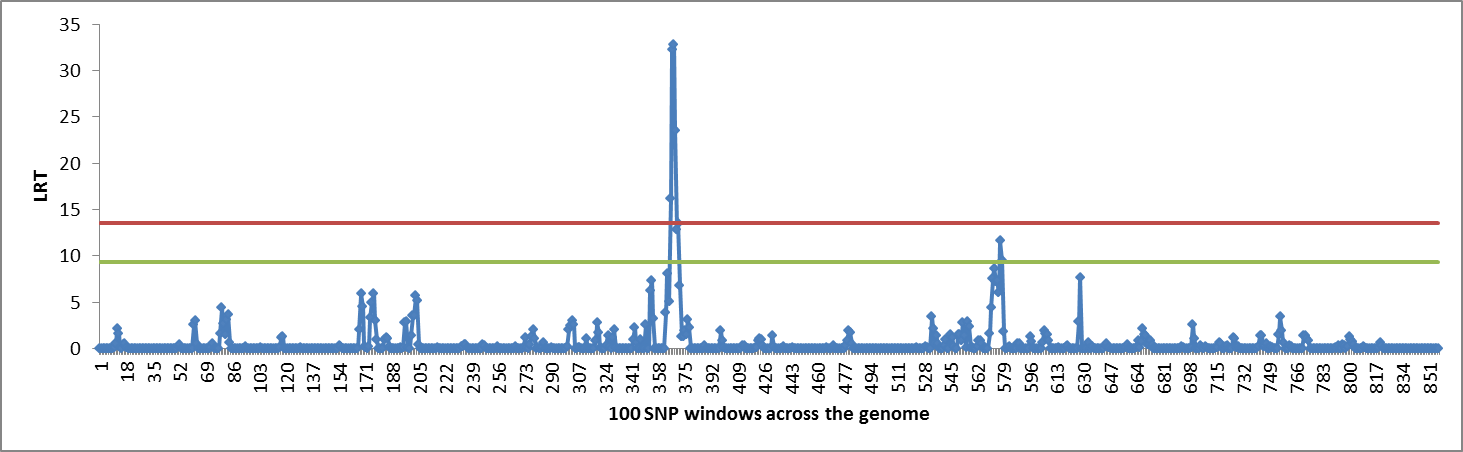


**Figure S20 Manhattan plot for fat density at the 8th thoracic vertebra accounting for live weight using Regional Heritability Mapping**


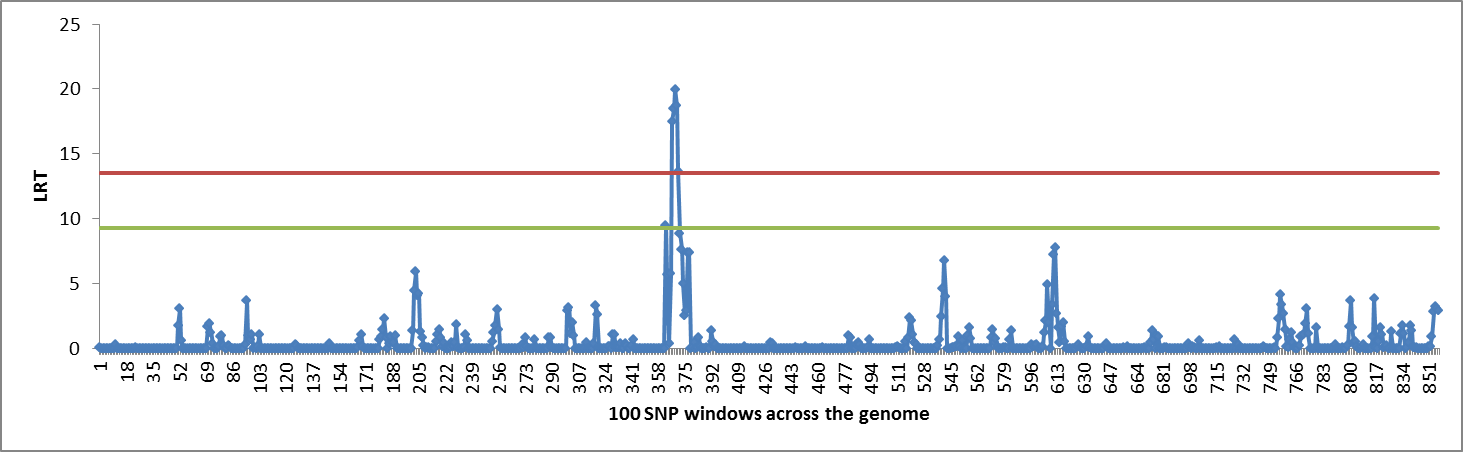

Supplement: Supplementary file 7 — 10.1186/s12711-016-0191-3 Genome-wide Manhattan plots for fat traits using regional heritability mapping. [file 12711_2016_191_MOESM7_ESM.docx]
