# Supplementary material for: Genome-wide association reveals QTL for growth, bone and in vivo carcass traits as assessed by computed tomography in Scottish Blackface lambs
Source: Genet Sel Evol. 2016 Feb 8;48:11. doi: 10.1186/s12711-016-0191-3 (PMC4745175; doi:10.1186/s12711-016-0191-3)

**Additional file 8**

**Figure S21 Manhattan plot for fat area at the ischium accounting for live weight using Regional Heritability Mapping**


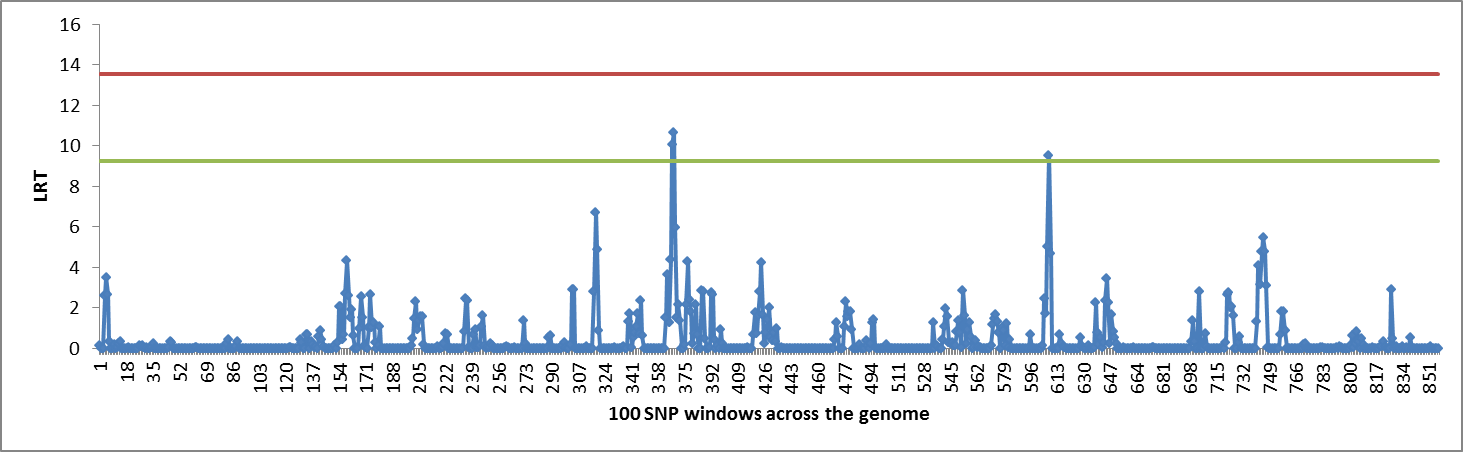


**Figure S22 Manhattan plot for fat area at the 8th thoracic vertebra using Regional Heritability Mapping**


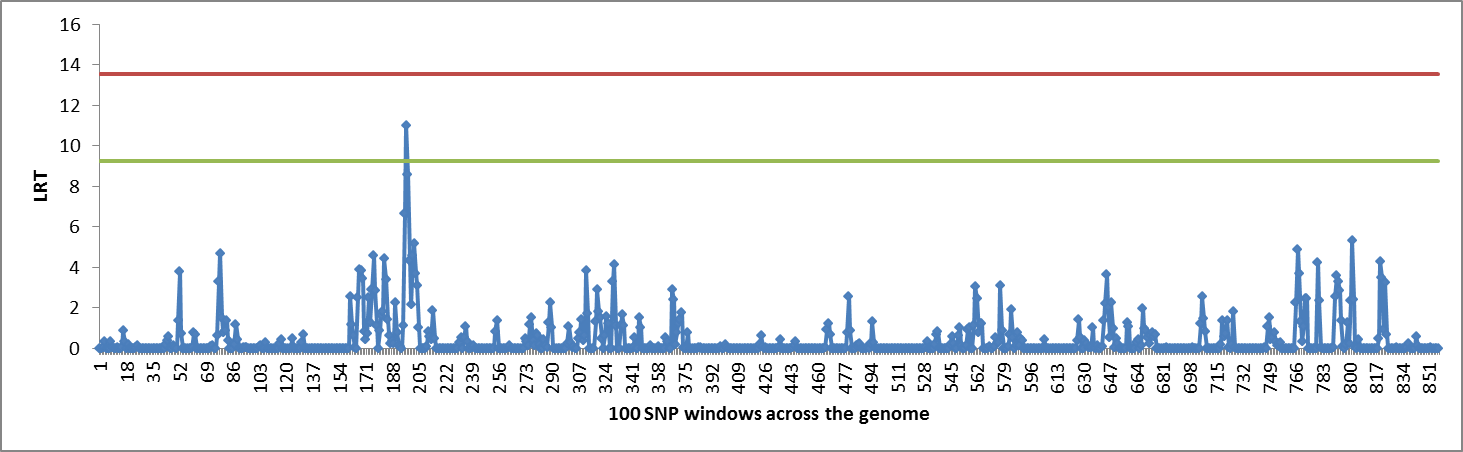


**Figure S23 Manhattan plot for fat density at the ischium accounting for live weight using Regional Heritability Mapping**


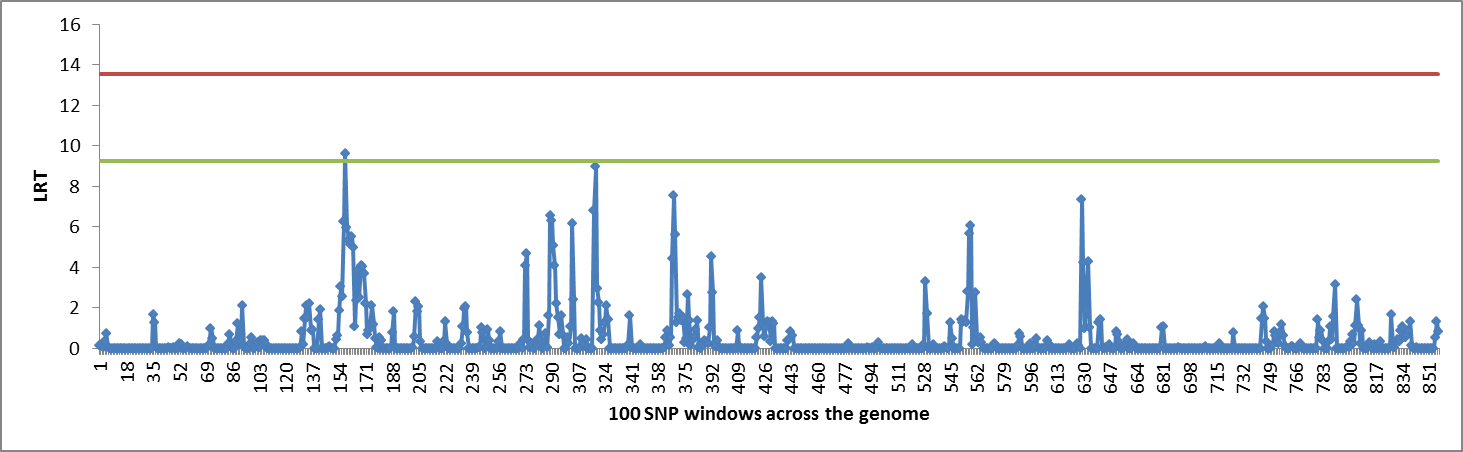


**Figure S24 Manhattan plot for muscle weight using Regional Heritability Mapping**


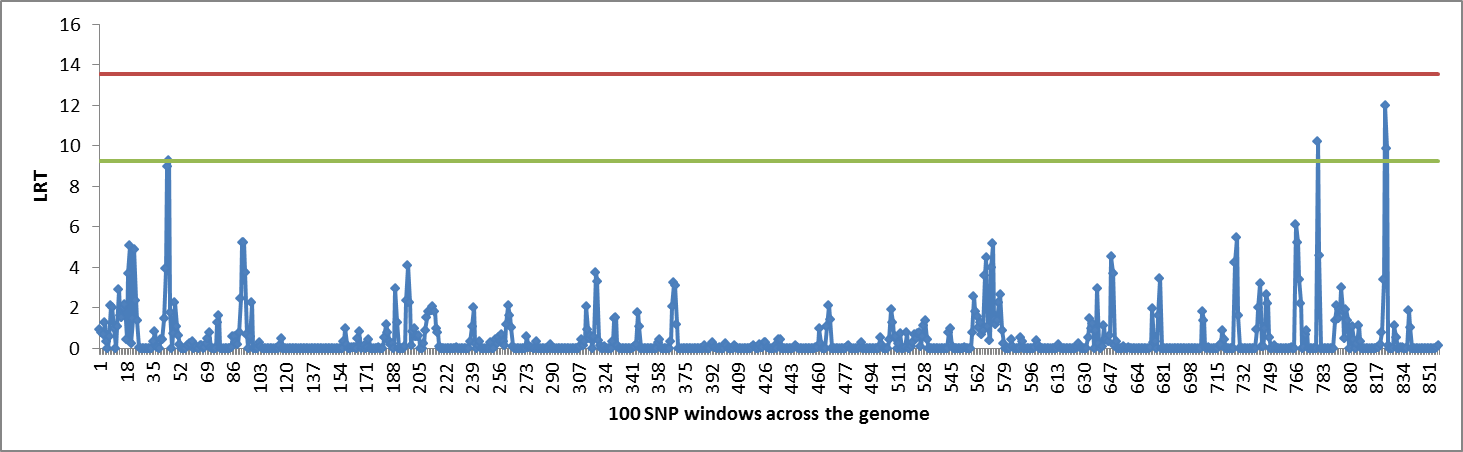


**Figure S25 Manhattan plot for muscle area at the ischium using Regional Heritability Mapping**


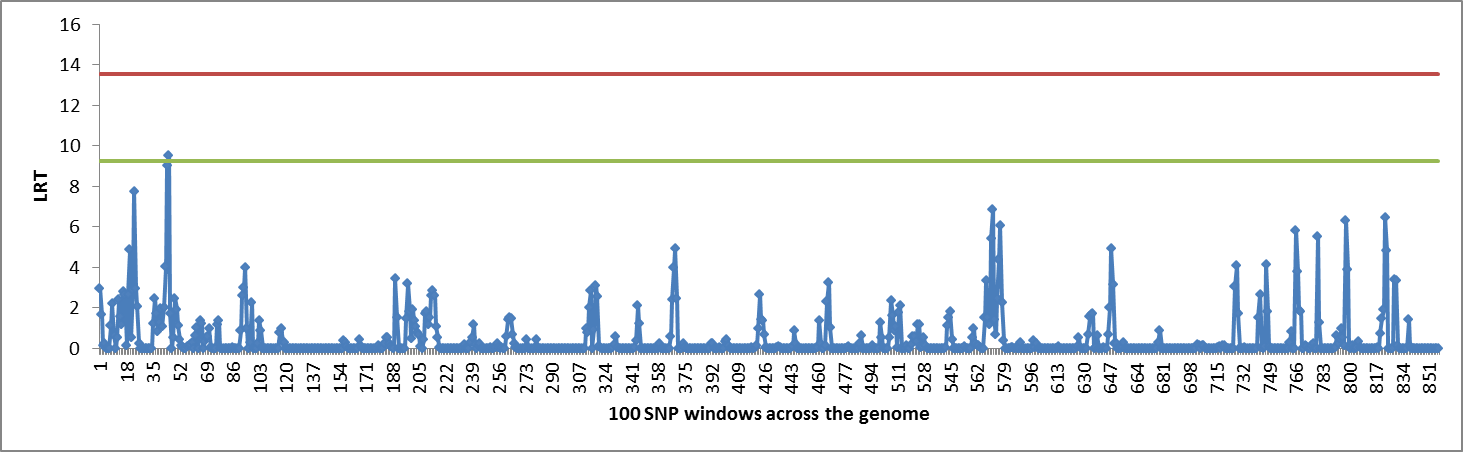


**Figure S26 Manhattan plot for muscle area at the 5th lumbar vertebra accounting for live weight using Regional Heritability Mapping**


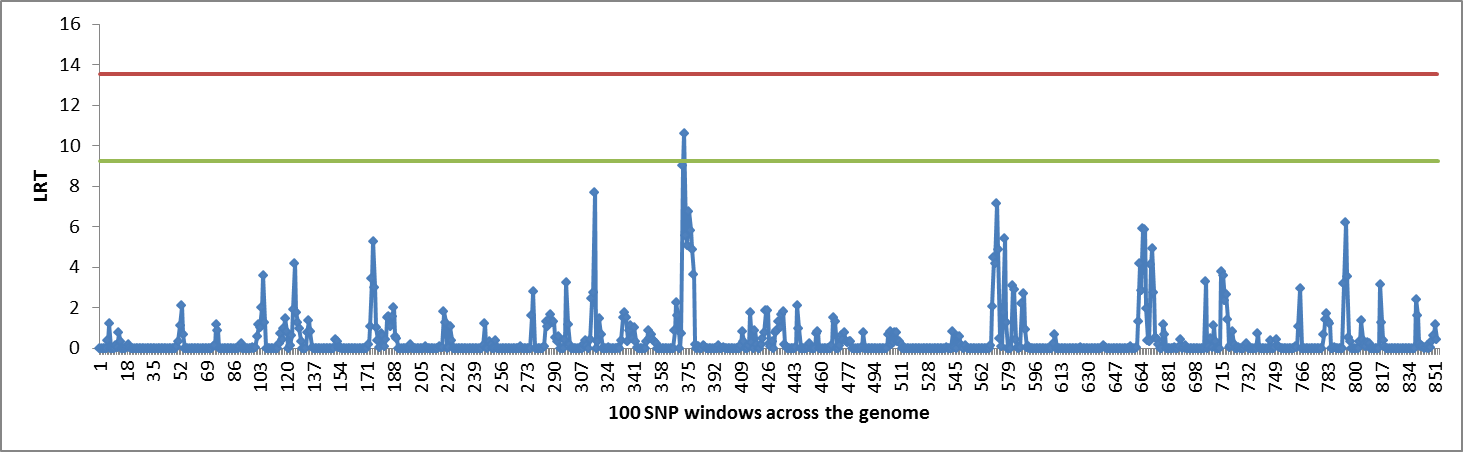


**Figure S27 Manhattan plot for muscle area at the 8th thoracic vertebra using Regional Heritability Mapping**


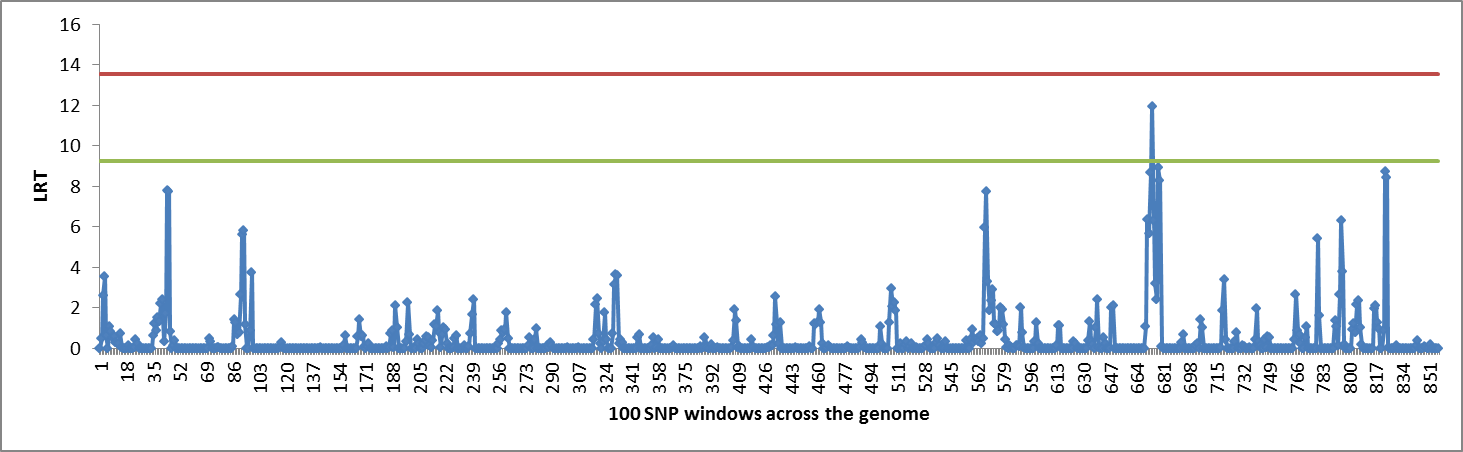


**Figure S28 Manhattan plot for muscle area at the 8th thoracic vertebra accounting for live weight using Regional Heritability Mapping**


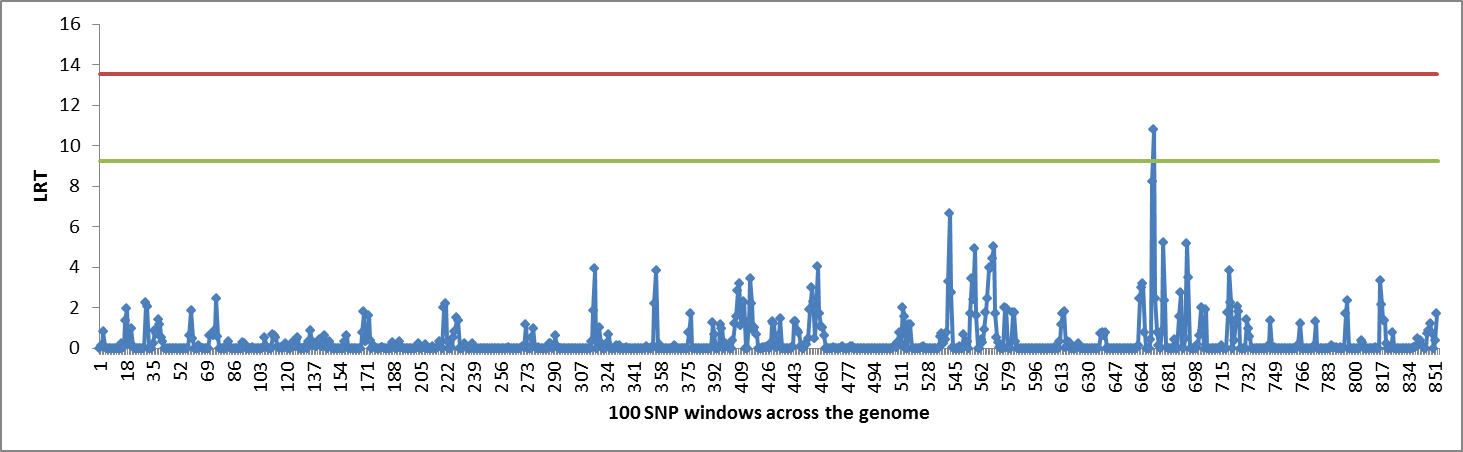


**Figure S29 Manhattan plot for muscle density at the ischium using Regional Heritability Mapping**


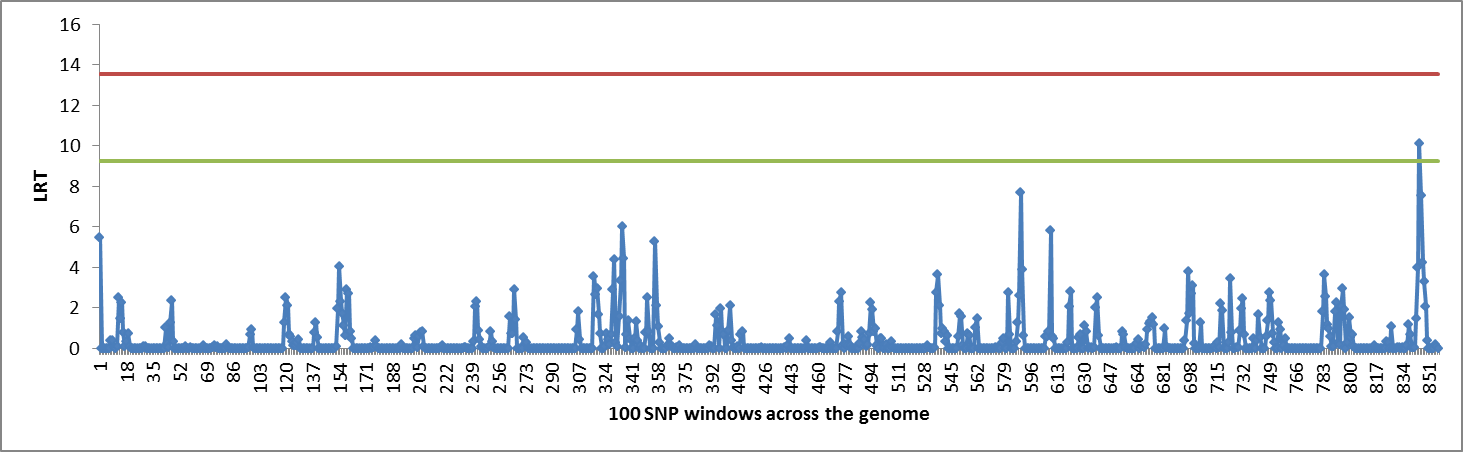


**Figure S30 Manhattan plot for muscle density at the ischium accounting for live weight using Regional Heritability Mapping**


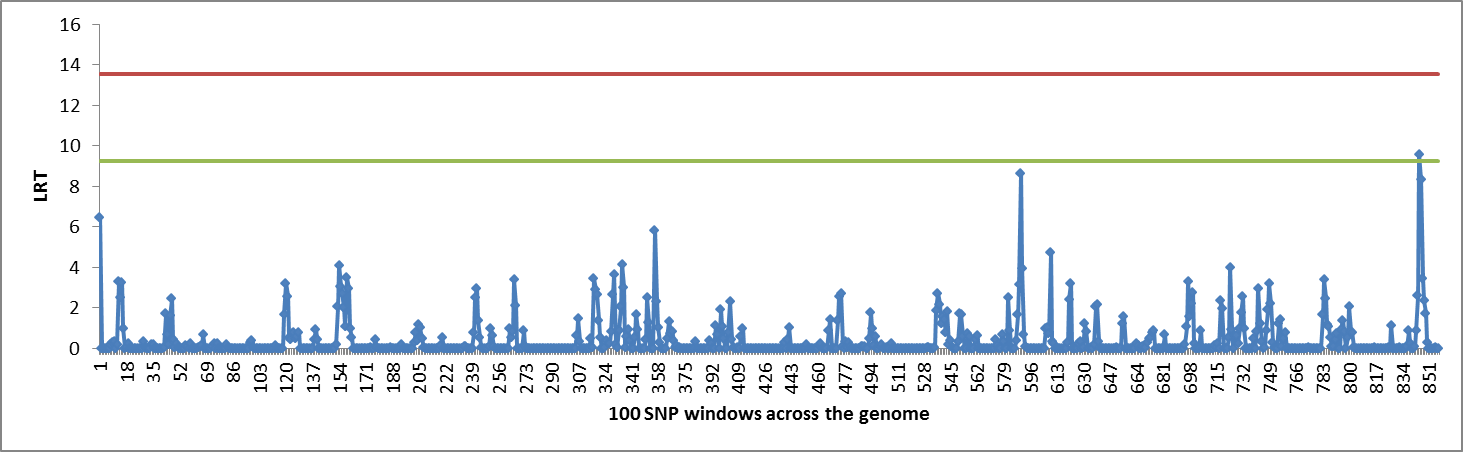


**Figure S31 Manhattan plot for muscle density at the 5th lumbar vertebra using Regional Heritability Mapping**


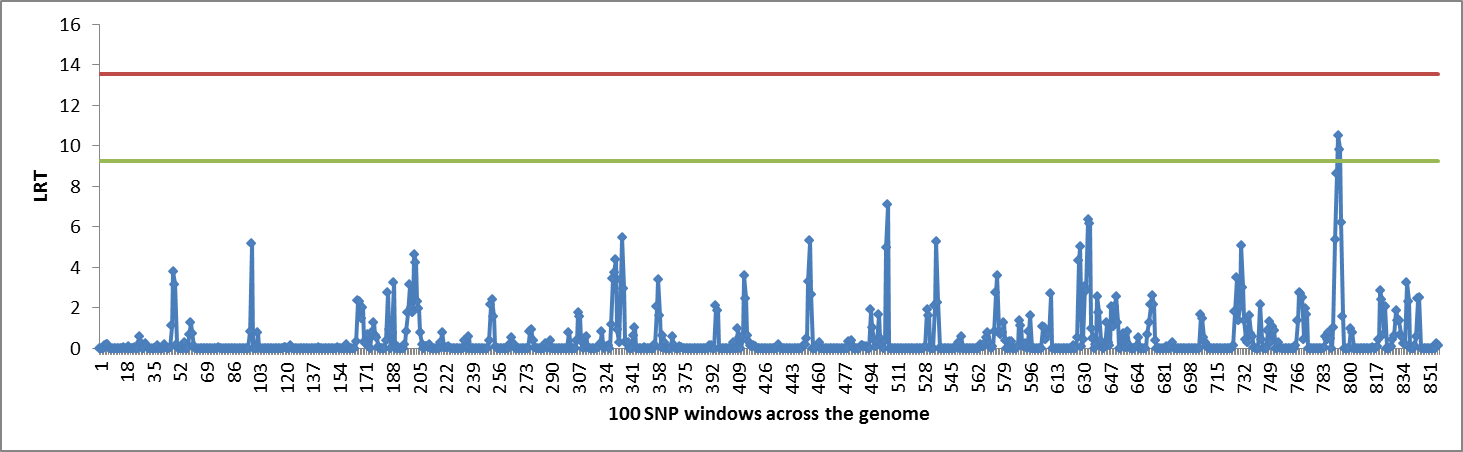


**Figure S32 Manhattan plot for muscle density at the 8th thoracic vertebra using Regional Heritability Mapping**


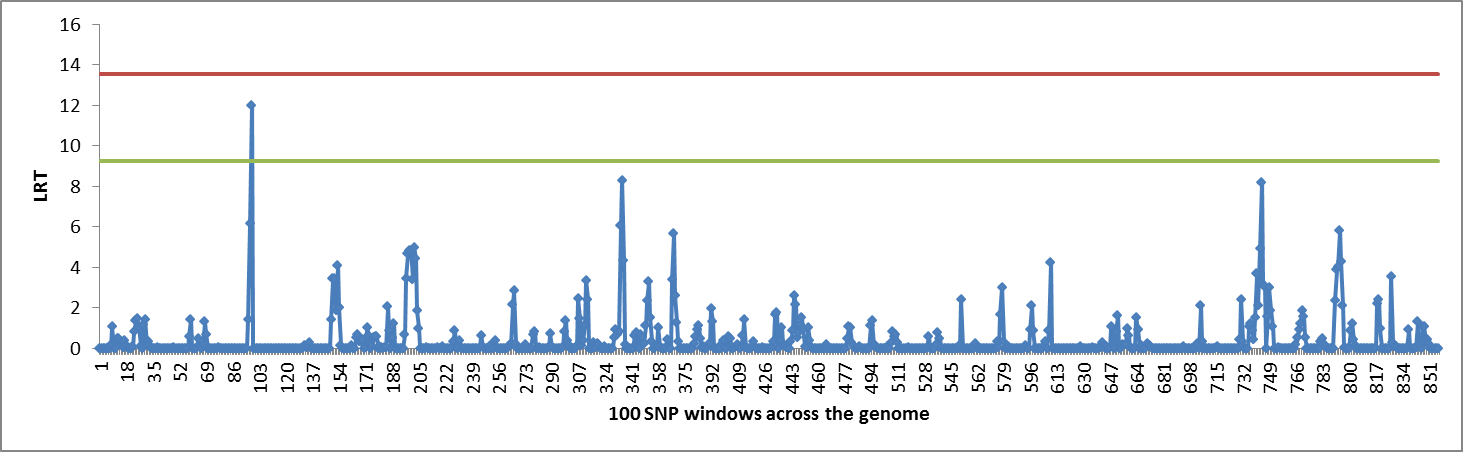


**Figure S33 Manhattan plot for bone proportion using Regional Heritability Mapping**


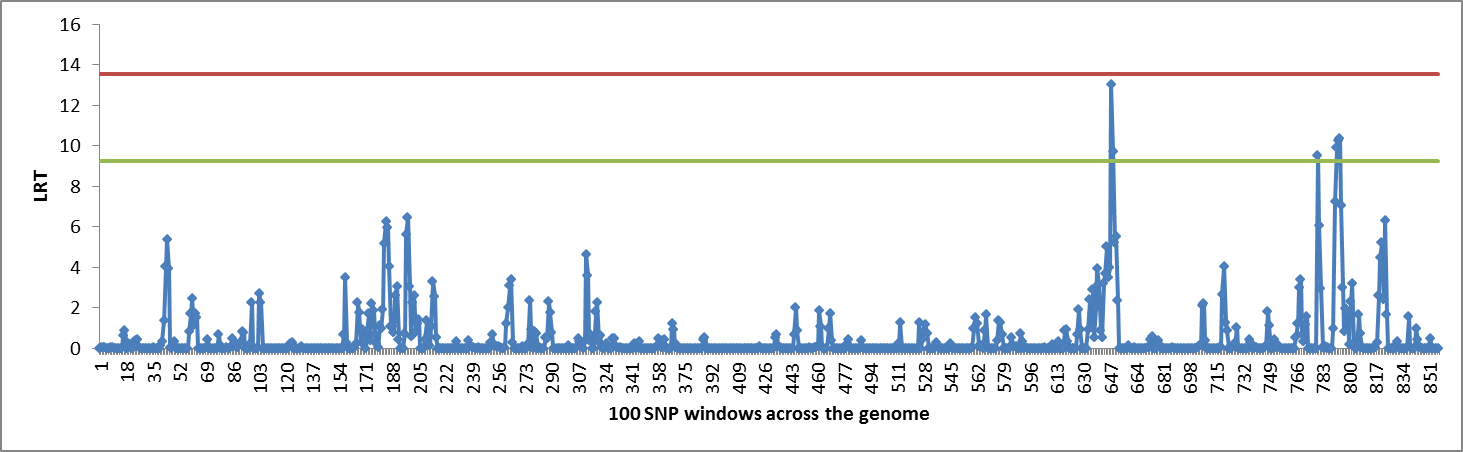


**Figure S34 Manhattan plot for total carcass weight using Regional Heritability Mapping**


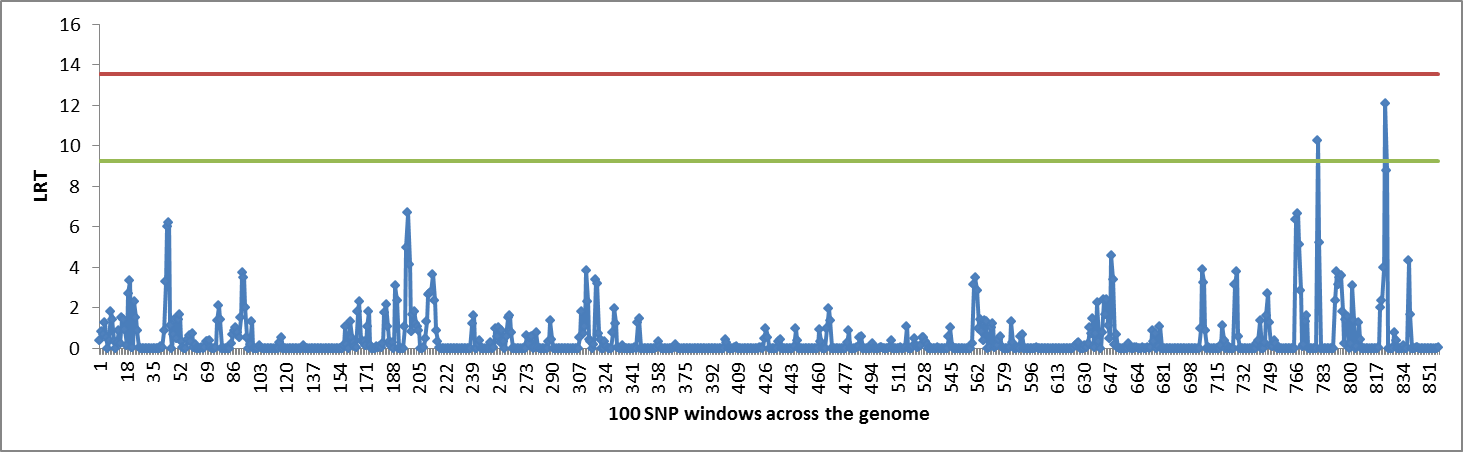


**Figure S35 Manhattan plot for muscle to bone ratio using Regional Heritability Mapping**


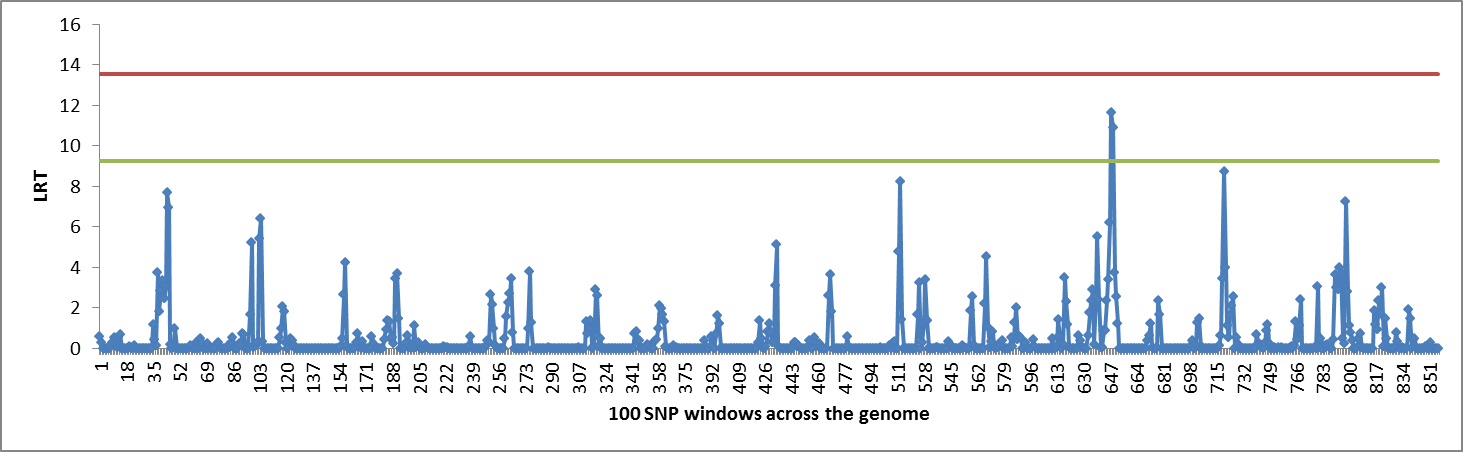


**Figure S36 Manhattan plot for live weight using Regional Heritability Mapping**


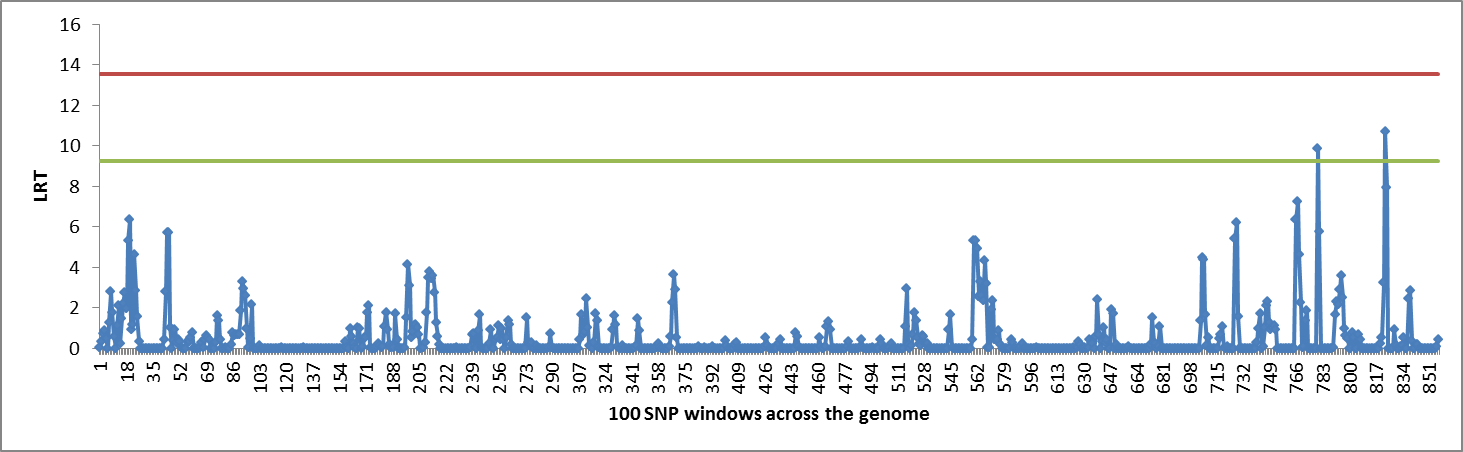

Supplement: Supplementary file 8 — 10.1186/s12711-016-0191-3 Suggestive Manhattan plots for fat, muscle and proportion traits using regional heritability mapping. [file 12711_2016_191_MOESM8_ESM.docx]
