# Supplementary material for: Genome-wide association reveals QTL for growth, bone and in vivo carcass traits as assessed by computed tomography in Scottish Blackface lambs
Source: Genet Sel Evol. 2016 Feb 8;48:11. doi: 10.1186/s12711-016-0191-3 (PMC4745175; doi:10.1186/s12711-016-0191-3)

**Additional file 9**

**Figure S37** Linkage disequilibrium (LD) values (r2 values, expressed as percentage) between SNPs within the interval ranging from SNP s38306 (at 35347156 bp) to SNP OAR6_41768532 (at 37533664 bp) on OAR 6. The colour scheme is as follows: white for r^2^ = 0, shades of grey for 0 < r^2^ < 1 and black for r2 = 1.


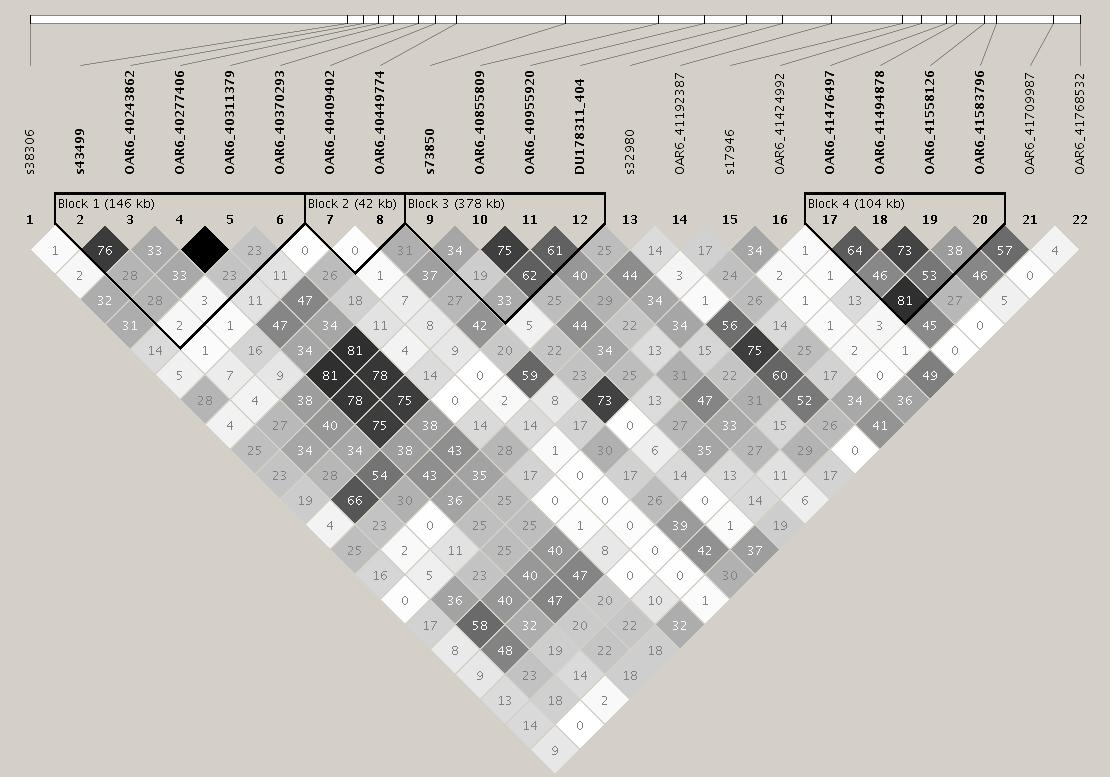


**Figure S38** D prime (D') values (D', representing allele frequencies) between SNPs in the interval ranging from SNP s38306 (at 35347156 bp) to SNP OAR6_41768532 (at 37533664 bp) on OAR 6. The standard colour scheme is as follows: white for low D' value (D' < 1) and low LOD value (LOD < 2), blue for high value D' (D' = 1), shades of pink/red for low D' value and low LOD value and bright red for high D' value and high LOD value.


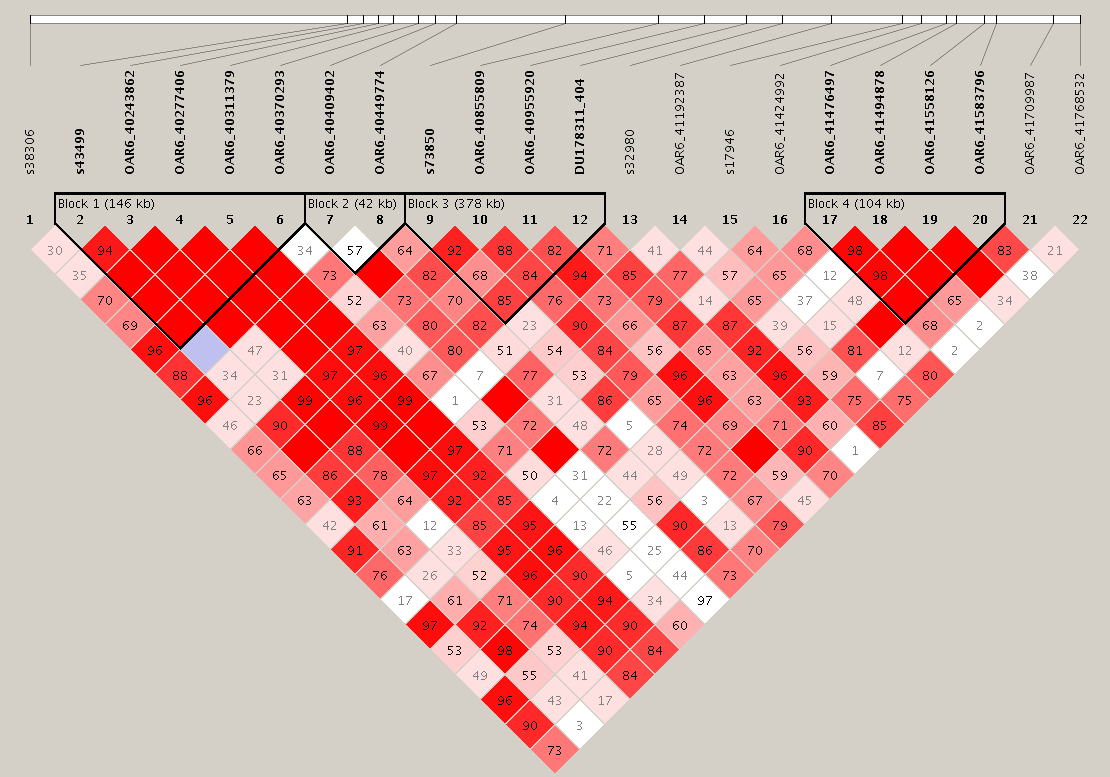


**Figure S39** Phased population haplotypes showing each haplotype in a block with the corresponding population frequency and connections from one block to the next one, including in the crossing areas, a value of multiallelic D' shown as a representation of the level of recombination between the two blocks.


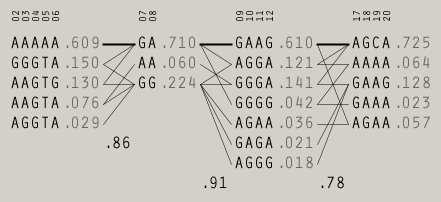

Supplement: Supplementary file 9 — 10.1186/s12711-016-0191-3 Linkage disequilibrium (r2 or D′ values) and phased population haplotypes within the region between 35,347,156 and 37,533,664 bp on OAR6. [file 12711_2016_191_MOESM9_ESM.docx]
